# A Novel Locus Harbouring a Functional *CD164* Nonsense Mutation Identified in a Large Danish Family with Nonsyndromic Hearing Impairment

**DOI:** 10.1371/journal.pgen.1005386

**Published:** 2015-07-21

**Authors:** Mette Nyegaard, Nanna D. Rendtorff, Morten S. Nielsen, Thomas J. Corydon, Ditte Demontis, Anna Starnawska, Anne Hedemand, Annalisa Buniello, Francesco Niola, Michael T. Overgaard, Suzanne M. Leal, Wasim Ahmad, Friedrik P. Wikman, Kirsten B. Petersen, Dorthe G. Crüger, Jaap Oostrik, Hannie Kremer, Niels Tommerup, Morten Frödin, Karen P. Steel, Lisbeth Tranebjærg, Anders D. Børglum

**Affiliations:** 1 Department of Biomedicine, Aarhus University, Aarhus, Denmark; 2 Centre for Integrative Sequencing (iSEQ), Aarhus University, Aarhus, Denmark; 3 Wilhelm Johannsen Centre for Functional Genome Research, Department of Cellular and Molecular Medicine (ICMM), The Panum Institute, University of Copenhagen, Copenhagen, Denmark; 4 Department of Otorhinolaryngology, Head & Neck Surgery and Audiology, Bispebjerg Hospital/Rigshospitalet, Copenhagen, Denmark; 5 Clinical Genetic Clinic, Kennedy Center, Copenhagen University Hospital, Rigshospitalet, Glostrup, Denmark; 6 Wolfson Centre for Age-Related Diseases, King's College London, London, United Kingdom; 7 Biotech Research & Innovation Centre (BRIC), University of Copenhagen, Copenhagen, Denmark; 8 Department of Chemistry and Bioscience, Aalborg University, Aalborg Ø, Denmark; 9 Center for Statistical Genetics, Department of Molecular and Human Genetics, Baylor College of Medicine, Houston, Texas, United States of America; 10 Department of Biochemistry, Faculty of Biological Sciences, Quaid-i-Azam University, Islamabad, Pakistan; 11 Department of Molecular Medicine, Aarhus University Hospital, Skejby, Aarhus, Denmark; 12 Department of Clinical Genetics, Vejle Hospital, Vejle, Denmark; 13 Department of Otorhinolaryngology, Radboud University Medical Center, Nijmegen, Nijmegen, Netherlands; 14 Department of Human Genetics, Radboud University Medical Center, Nijmegen, Nijmegen, Netherlands; 15 Radboud Institute for Molecular Life Sciences, Radboud University Medical Center, Nijmegen, Nijmegen, Netherlands; University of Michigan, UNITED STATES

## Abstract

Nonsyndromic hearing impairment (NSHI) is a highly heterogeneous condition with more than eighty known causative genes. However, in the clinical setting, a large number of NSHI families have unexplained etiology, suggesting that there are many more genes to be identified. In this study we used SNP-based linkage analysis and follow up microsatellite markers to identify a novel locus (DFNA66) on chromosome 6q15-21 (LOD 5.1) in a large Danish family with dominantly inherited NSHI. By locus specific capture and next-generation sequencing, we identified a c.574C>T heterozygous nonsense mutation (p.R192*) in *CD164*. This gene encodes a 197 amino acid transmembrane sialomucin (known as endolyn, MUC-24 or CD164), which is widely expressed and involved in cell adhesion and migration. The mutation segregated with the phenotype and was absent in 1200 Danish control individuals and in databases with whole-genome and exome sequence data. The predicted effect of the mutation was a truncation of the last six C-terminal residues of the cytoplasmic tail of CD164, including a highly conserved canonical sorting motif (YXXФ). In whole blood from an affected individual, we found by RT-PCR both the wild-type and the mutated transcript suggesting that the mutant transcript escapes nonsense mediated decay. Functional studies in HEK cells demonstrated that the truncated protein was almost completely retained on the plasma cell membrane in contrast to the wild-type protein, which targeted primarily to the endo-lysosomal compartments, implicating failed endocytosis as a possible disease mechanism. In the mouse ear, we found CD164 expressed in the inner and outer hair cells of the organ of Corti, as well as in other locations in the cochlear duct. In conclusion, we have identified a new DFNA locus located on chromosome 6q15-21 and implicated *CD164* as a novel gene for hearing impairment.

## Author Summary

It is known that hearing impairment running in families can be caused by mutations in more than eighty different genes. However, there are still families where the responsible gene is unknown. By studying a large Danish family with dominant inherited hearing impairment, we found that the disorder cosegregates with genetic markers on chromosome 6, suggesting that the responsible mutation lies within this chromosomal region. By sequencing this genetic locus, we discovered a mutation in the CD164 gene that is passed on to all the affected individuals. In the mouse ear, we demonstrated that the CD164 protein is expressed in hair cells and other sites known to be important for correct hearing. The identified mutation is predicted to result in shortening of the protein, leading to loss of an evolutionary conserved sequence important for cellular trafficking of CD164. Using cell lines, we show that the truncated protein is trapped on the cell surface while the normal protein is internalized. This finding is important because it implicates for the first time a role for CD164 in the complex physiological processes of hearing and suggests that failed endocytosis may be a possible disease mechanism for some types of hearing impairment.

## Introduction

Nonsyndromic hearing impairment (NSHI) is the most frequent hereditary sensory defect in humans worldwide. The condition is clinically and genetically extremely heterogeneous, with more than 160 loci identified today. Autosomal dominant NSHI (ADNSHI) shows great variation in age of onset, rate of progression, severity and frequencies affected in contrast to autosomal recessive NSHI (ARNSHI) that is usually congenital/prelingual and non-progressive [1].

Currently, around 30 causative genes for ADNSHI have been identified. These genes are involved in a wide variety of molecular processes such as gene regulation, cytoskeleton dynamics, cell-cell junction formation, endocytosis and membrane transport [2]. Additional causative genes are expected to be discovered, since over 20 loci have been mapped without the corresponding genes being identified, and novel loci and/or genes are regularly being uncovered (http://hereditaryhearingloss.org) [1,3].

In the clinical field, identification of these hearing loss genes has greatly aided genetic counselling on hearing impairment. With the advances in next-generation sequencing technologies it is now possible to quickly screen most known genes implicated in NSHI simultaneously either by using customized capture arrays for targeted genes or exome sequencing [3,4] for the benefit of families, where the causative mutation can be identified. For these cases, diagnosis as well as important predictive information for the remaining family members can be offered [5]. However, with the extreme genetic heterogeneity in NSHI, a large proportion of the screened families still have an unexplained etiology.

In this study, we identified a novel locus (DFNA66) for dominant inherited NSHI on 6q15-21 in a large Danish family. By the use of a custom capture array and next-generation sequencing, we searched for the causative mutation in the region and identified a nonsense mutation in *CD164* [OMIM 603356]. The gene encodes CD164, a small transmembrane sialomucin protein involved in adhesion, migration and endocytosis and we provide data on the variant-, gene-, and functional level implicating the gene in hearing impairment.

## Results

### Linkage of hearing impairments to chromosome 6q15-21 in a large Danish family

**Fig 1:**
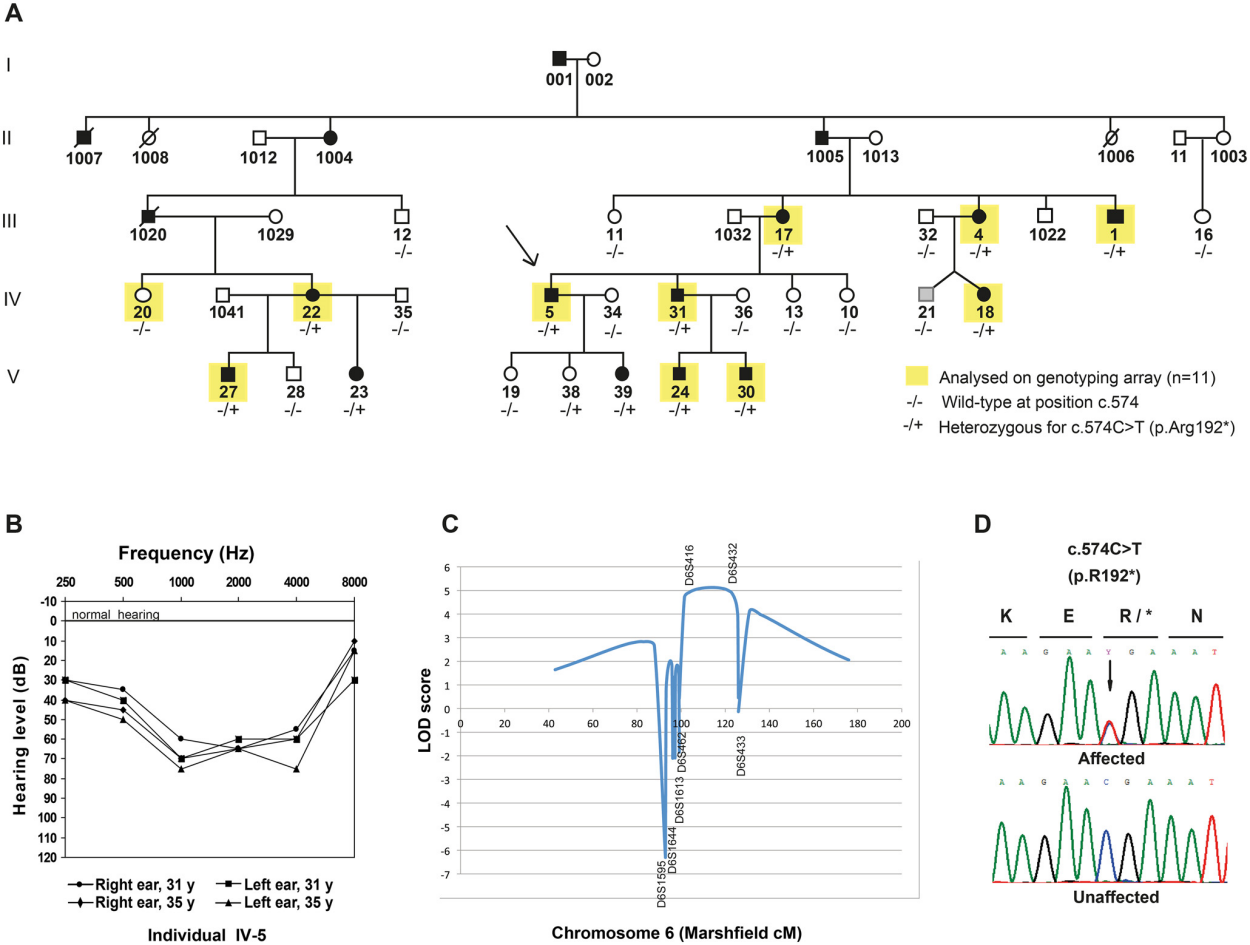
Pedigree, audiograms and linkage peak for a novel locus for dominant inherited nonsyndromic hearing impairment (NSHI). (A) Pedigree of a large Danish family with moderate hearing impairment, with the proband indicated with an arrow. DNA was available from all individuals except those with a four-digit ID. The presence (+) or absence (-) of the *CD164* mutation c.574C>T (p.R192*) is listed underneath each individual. The phenotype of individual (IV-21) was set to unknown (shown in grey) because of uncertainties about the origin of his hearing loss. (B) Audiograms of left and right ear of a representative affected family members (individual IV-5). Mid-frequencies are more severely affected than lower and higher frequencies termed basin shaped or cookie bite hearing loss. The age of the individual at the time of each analysis is indicated. (C) Genome-wide significant linkage to chromosome 6q15-21 was identified in an initial SNP-array analysis including 11 individuals (indicated in yellow, Fig 1A). (D) Chromatograms of the c.574C>T mutation in *CD164* exon 6 in an affected family member compared to a healthy control individual. Nomenclature refers to RefSeq NM_006016.4 (*CD164* isoform 1), with nucleotide number +1 being A of the start codon ATG.

A multi-generational family from Denmark with ADNSHI, affecting 17 individuals in five generations (Fig 1A) participated in the study. Audiograms and audiological data were collected from 13 individuals born between 1931 and 2003. The hearing impairment is moderate to severe (Fig 1B). Age of onset varied from newborn (detected through neonatal screening), age 3–6 or early twenties. The audiograms showed variable patterns with either a flat audiogram affecting all frequencies, or, at least initially, a basin shape with the most severe affection on the mid-frequencies. In some cases the hearing impairment remained stable, in others it progressed somewhat affecting a broader spectrum of frequencies over the years. Representative audiograms can be found in S1 Fig. One family member (IV-21, Fig 1A) with hearing impairment had experienced severe recurrent otitis media in childhood. From careful assessment of his audiograms (S1 Fig) we were not able to unequivocally determine if his hearing impairment was conductive or sensorineural. His phenotype was therefore set to unknown (grey pedigree symbol, Fig 1A).

Initial sequencing of seven known hearing loss genes (*WFS1* [OMIM 606201], *GRHL2* [OMIM 608576], *EYA4* [OMIM 603550], *ACTG1* [OMIM 102560], *GJB2* [OMIM 121011], *MYO6* (exon 25) [OMIM 600970], and *SLC26A4* [OMIM 605646]) failed to identify any mutations, prompting us to perform a genome-wide linkage analysis to identify the responsible locus for the hearing impairment in the family. Eleven individuals were then selected for single nucleotide polymorphism (SNP) genotyping using the Affymetrix 50K Xba240 array. The genotyped individuals are indicated with yellow squares in Fig 1A. After quality control and SNP pruning, 11,034 markers in approximate linkage equilibrium were included in a parametric linkage analysis using an autosomal dominant model with full penetrance and allele frequencies obtained from the CEU population. A single 25 Mb genome-wide significant linkage peak was identified on chromosome 6q15-q21 (LOD score = 3.6), with the critical haplotype flanked by markers rs9294390 (88,556,380 bp) and rs6910441 (113,518,576 bp) (hg19) (S1 Table). This region contains 101 annotated genes (S2 Table). The locus is relatively close to *EYA4* (DFNA10) [6], however a meiotic cross-over in three affected individuals excluded DFNA10 as the cause of the hearing loss in this family, consistent with the initial sequencing where no variations were found in *EYA4*.

To validate and possibly narrow down the locus, 26 family members were genotyped for seven microsatellite markers across the locus. A multipoint linkage analysis was carried out with allele frequencies determined from all genotyped founders and penetrance set to 1. In the analysis, the affection status was set to “unknown” for three individual in total. These were IV-21, because of the uncertainty about the origin of his hearing impairment (see description of the family) and individual V-19 and V-38 because of their young age (16 and 10 years respectively) being below the upper observed age of onset of the hearing impairment in this family. The analysis including 23 individuals mapped the locus between D6S462 (90,928,511 bp) and D6S433 (first marker outside region), thus narrowing down the locus by approximately 2 Mb in the proximal end and increasing the LOD score to 5.1 (Fig 1C). The genomic position of the locus is 90,928,511 to 113,518,576 bp (hg19).

### Next-generation sequencing and identification of a CD164 nonsense mutation

In an attempt to identify the causal mutation, nine candidate genes within the linked region were Sanger sequenced: *SOBP* [OMIM 613667] and *FOXO3* [OMIM 602681], known to cause deafness in mice [7,8], and seven other genes (*GJA10* [OMIM 611924], *POU3F2* [OMIM 600494], *FAXC* (also known as *C6orf168)* [no OMIM], *LIN28B* [OMIM 611044], *Hsa-mir-587* [no OMIM], *AMD1* [OMIM 180980], and *LAMA4* [OMIM 600133]), selected based on homology to known hearing loss genes or expression in the inner ear. Only common sequence variations (MAF above 1% in ESP6500), unlikely to cause hearing loss, were identified in these genes. We then applied a NimbleGen customized targeted capture array and next-generation sequencing (NGS) in order to sequence the entire locus in one affected individual (IV-31) (Fig 1A). Statistics for the bioinformatics analysis can be seen in S4 Table. After uploading the VCF file to Ingenuity Variant Analysis, an initial filtering based on mapping quality and chromosomal position identified 28,200 variations across the entire locus. After filtering out common variants (MAF above 1%), 1609 variants remained. Of these, two were found in coding regions; a variant c.574C>T [NM_006016.4] in *CD164* and rs143143212 in *MMS22L*. Filtering the 1609 variants for functional effect (S2 Fig), one variant passed through the filter i.e. c.574C>T in *CD164*. The variant is predicted to cause a truncation of CD164 by introducing a premature stop codon at amino acid position 192 (p.R192*) [UniProtKB NP_006007.2]) and is not present in any available databases. Genotyping of all 26 family members with DNA available confirmed that the *CD164* mutation was found in all individuals carrying the critical haplotype (Fig 1A and 1D). Genotyping of 1200 unrelated Danish control individuals for the c.574C>T nonsense mutation did not identify anyone carrying the c.574C>T variant. By genotyping 2400 control chromosomes from the same background population as the family, the power is 80% to detect a variant with a minor allele frequency as low as 0.001, suggesting that the mutation is unlikely to be a rare polymorphism in the Danish population.

To ask if *other* nonsense or frameshift mutations in *CD164* had been reported, we searched all relevant, available databases. In dbSNP138, we found a nonsense mutation (rs11542733) which was originally submitted to dbSNP120 by a large-scale sequencing effort of expressed sequence tags in 2001 [9]. The mutation was reported in individual NA06993 (CEPH 1341.13). We obtained genomic DNA from this individual (Coriell Cell Repositories, New Jersey, USA) and by Sanger sequencing we were not able to confirm the presence of this mutation (S3 Fig), suggesting that the record is likely due to an artefact from early high throughput sequencing. In conclusion, the c.574C>T mutation is to our knowledge the first *CD164* nonsense mutation identified in humans.

To estimate the frequency of *CD164* mutations among patients with unknown cause of hearing impairment, we sequenced all coding exons and splice junctions of *CD164* using DNA samples from 46 independent index cases. The cases were 15 unrelated probands from Denmark (the index patient from 12 families and 3 sporadic cases) selected based on their hearing impairment phenotype with basin shaped audiograms, 25 index patients from the Netherlands based on phenotype with postlingual onset (1^st^ or 2^nd^ decade), progression of the hearing impairment and cookie-bite or flat audiogram configuration, and 6 probands of Pakistani families with ARNSHI that displayed linkage to chromosome 6. The recessive families were included as several hearing impairment genes (*e*.*g*. *TMC1* [OMIM 606706], *TECTA* [OMIM 602574], *MYO7A* [OMIM 276903]) have been found to underlie both autosomal-dominant and recessive NSHI (http://hereditaryhearingloss.org/). However, no sequence variants likely to cause hearing impairment were found, suggesting that mutations in *CD164* are not a common cause of NSHI.

**Fig 2:**
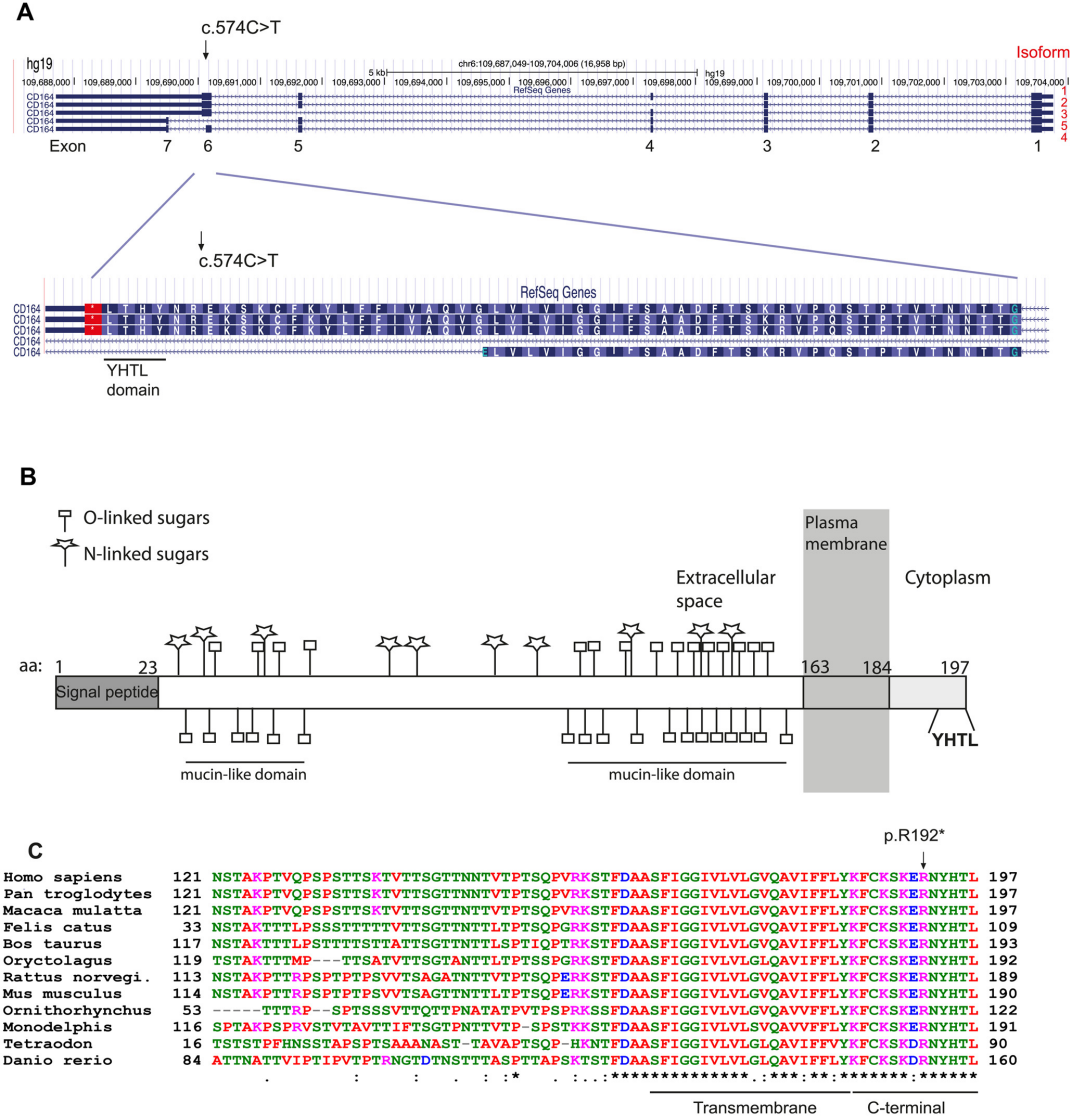
Isoforms of *CD164* and evolutionary conservation of the C-terminal YHTL sorting motif. (A) The nonsense mutation was identified in exon 6, used by the three membrane bound isoforms of CD164. (B) A schematic representation of CD164 isoform 1. The protein contains two mucin-like domains separated by a cysteine-rich domain [29]. The locations of potential N-glycosylations sites and predicted O-glycosylation sites were predicted from the NetOGlyc 4.0 Server (http://www.cbs.dtu.dk/services/NetOGlyc/). The location of the transmembrane region was predicted from the SMART database (smart.embl-heidelberg.de). (C) Alignment of the amino acid sequence of CD164 different species shows high evolutionary conservation of the C-terminal region including the YHTL sorting motif, which is deleted as a consequence of the nonsense mutation. Isoform 4 and 1 are the most predominantly expressed isoforms across a broad range of tissues. Information on isoforms was accessed from the GTEx Portal (http://www.gtexportal.org/home/).

*CD164* contains seven coding exons and expresses a protein referred to as CD164, MUC-24 or endolyn [10]. Five splice variants of the gene have been reported, with isoforms 1–3 encoding a membrane bound form by the use of the full exon 6, and isoforms 4 and 5 encoding a soluble form of the protein by alternative splicing of exon 6 or the alternative use of exon 7 (Fig 2A). Isoform 1 (ENST00000413644) and 4 (ENST00000310786) account for the vast majority of expressed transcripts across different tissues, found by the Genotype-Tissue Expression project (GTEx) [11]. As the c.574C>T mutation is located at the end of exon 6, the mutation is predicted to affect only the membrane bound forms of CD164 (isoforms 1–3). Isoform 1 encodes a 197 amino acid long protein with a large extracellular region with two heavily glycosylated mucin-like domains, separated by a cysteine-rich domain, a transmembrane domain, and a short cytoplasmic region containing a canonical YXXФ sorting motif (where X stands for any residue and Ф for a large hydrophobic residue) (YHTL) (Fig 2B). As previously mentioned, the c.574C>T mutation causes a substitution of an arginine (R192) for a stop codon (p.R192*), thereby deleting the last six amino acids of the CD164 C-terminus (RNYHTL), including the sorting motif. An amino acid sequence alignment of CD164 from different species shows a 100% conservation of these six C-terminal CD164 residues from human to roundworm (Fig 2C), indicating a high selective pressure against amino acid changes in this sequence, consistent with its role in subcellular trafficking of proteins to the lysosomal compartment in cells [12].

### The p.R192 mutation causes abnormal trafficking of CD164

**Fig 3:**
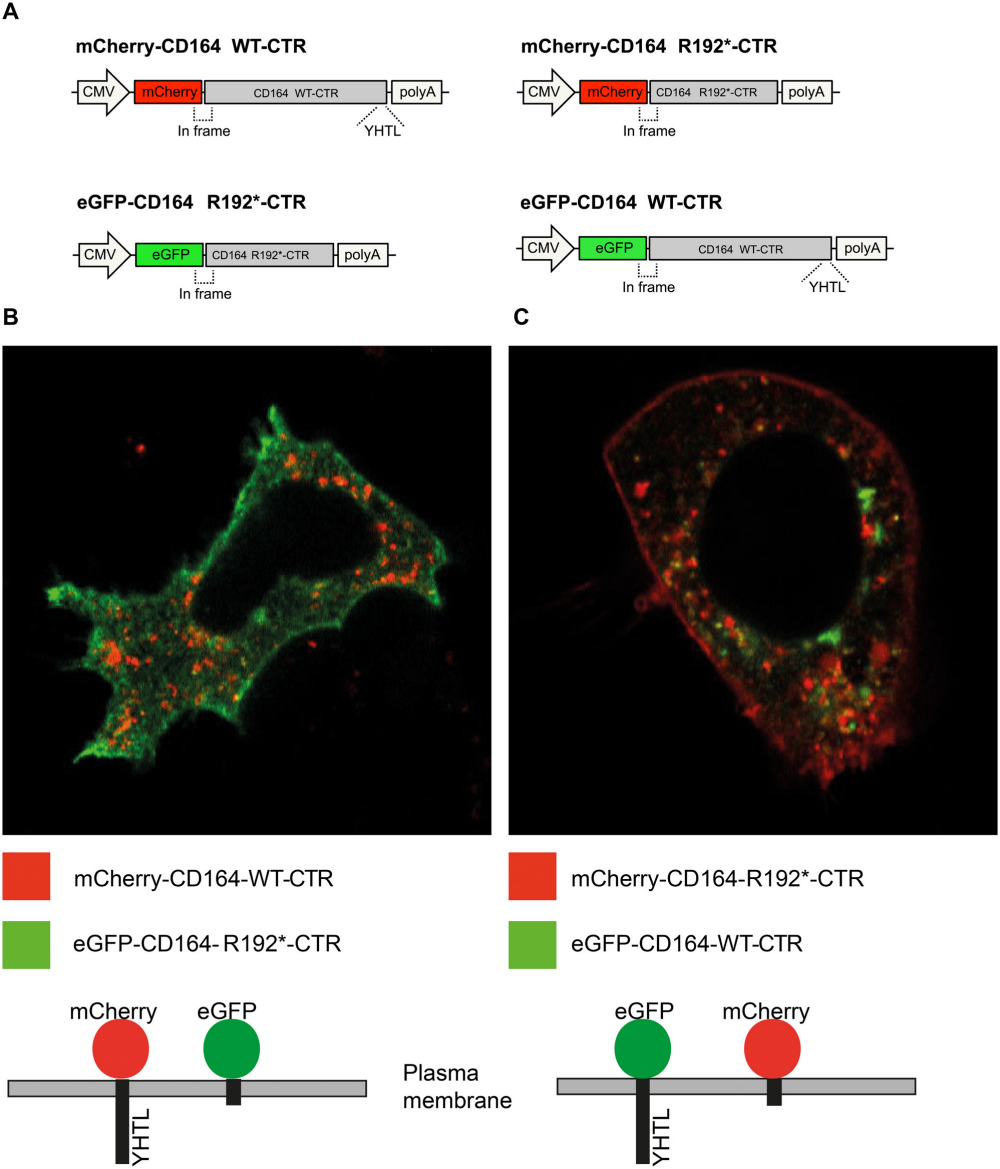
Membrane localization of fluorescent proteins fused to the CD164 C-terminal domain containing the R192* mutation. HEK-293 cells were co-transfected with plasmid expressing mCherry fused to the C-terminal region (CTR) of wild-type CD164 and eGFP fused to the CTR of CD164-R192* or *vice versa*. Forty-eight hours post-transfection, live imaging of the cells was performed using a confocal laser scanning microscope using 63×water-immersion objective. (A) Schematic of the constructs (B) Wild-type fusion protein (mCherry-CD164-WT-CTR) (red) was intracellularly located while the truncated fusion protein (eGFP-CD164-R192*-CTR) was primarily present at the plasma membrane. (C) Same result was found when reversing mCherry and eGFP (colour swap).

To assess the functional effect of the truncating mutation on sorting and localization of CD164, we first studied the subcellular localization of the *C-terminal region* (CTR) of wild-type and mutant CD164 fused to fluorescent marker proteins. We co-transfected human embryonic kidney (HEK)-293 cells with plasmids encoding two fusion proteins: (i) an mCherry fluorescent protein N-terminally fused to the transmembrane segment and the CTR of CD164 (mCherry-CD164-WT-CTR) and (ii) an eGFP fluorescent protein N-terminally fused to the transmembrane segment and the CTR of CD164 lacking the last 6 amino acids (eGFP-CD164-R192*-CTR) (Fig 3A). This was done to detect and distinguish the subcellular localization of wild-type and truncated CD164 C-terminal regions simultaneously in the same experiment. Using confocal microscopy, images of live cells were captured two days after transfection. This demonstrated that in the steady-state, the truncated fusion protein (green) was found mostly at the plasma membrane, while the wild-type fusion protein (red) was predominantly located in intracellular vesicles, suggesting a grossly abnormal sorting of the truncated fusion protein (Fig 3B). Identical findings were obtained when cells were transfected with plasmids encoding the opposite combination of fluorescent marker proteins (colour swap) (Fig 3C). In both dye swap experiments a small amount of truncated fusion protein was detected in the cytosol. Passive internalization is the most likely explanation for this because the truncated fusion protein was present at very high levels in the plasma membrane.

**Fig 4:**
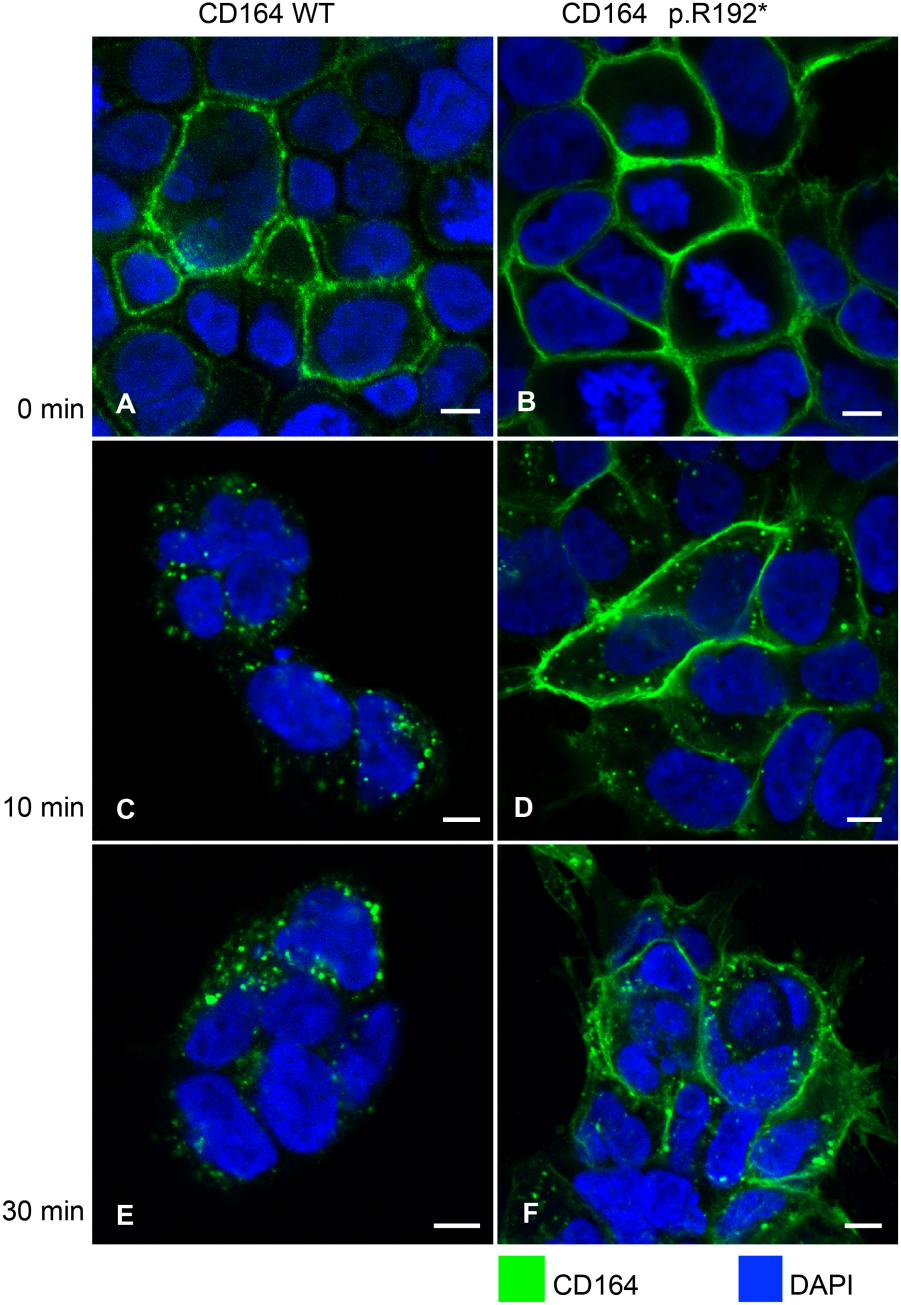
Failure of internalization of CD164 R192*. HEK cells stably overexpressing wild-type CD164 (CD164 WT) (A, C, E), and truncated CD164 (CD164 R192*) (B, D, F) were seeded on cover slides and were incubated with anti-CD164 antibody on ice. Next, cells were either fixed (T0, 0 min) or incubated at 37°C in complete medium without antibody for 10 (T10) and 30 (T30) minutes, respectively, and then fixed. Finally, CD164 was visualized using Alexa Fluor 488-labeled secondary antibody (green). Nuclear DNA was stained with DAPI (blue). Imaging was performed on a confocal laser scanning microscope using 40×oil-immersion objective. Scale bar = 6 μm.

To investigate if wild-type and R192* CD164 with intact extracellular domain would exhibit a similar trafficking difference as the C-terminal region, HEK cells were stably transfected with constructs encoding human full-length wild-type CD164 and the truncated CD164 R192*, respectively. A qPCR assay, able to distinguish wild-type and mutant transcripts and quantifying total CD164, were used to select two cell lines expressing wild-type and mutant CD164, respectively, at comparable levels (S3 Table and S4 Fig). The assay showed that endogenous CD164 expression in the mutant cell line accounting for around 20% of the total CD164 expression. Due to the high amount of CD164 (>95%) in the endo-lysosomal system under normal steady-state conditions, and in order to observe the timing of the endocytic trafficking of wild-type and mutant proteins, all CD164 present at the cell surface on living transfectants were saturated with anti-CD164 antibodies at 0°C, as cooling arrests internalisation (T0). At T0, CD164 was present at the plasma membrane in both cell lines, as expected (Fig 4A and 4B). The fate of CD164 was then followed after raising the temperature to 37°C to initiate internalization. After 10 minutes, most of the wild-type CD164 was internalized (Fig 4C) with no further change in localization after 30 min (T30) (Fig 4E), indicating that wild-type CD164 was rapidly (within minutes) cleared from the cell surface and that no recycling of CD164 took place within this timeframe. In contrast, only low levels of CD164 R192* were internalized after 10 and 30 minutes (Fig 4D and 4F). Untransfected HEK cells did not produce a CD164 signal over background in these stainings. This experiment demonstrated that CD164 R192* was trapped at the plasma membrane.

### CD164 and CD164 R192* can form heterodimers

**Fig 5:**
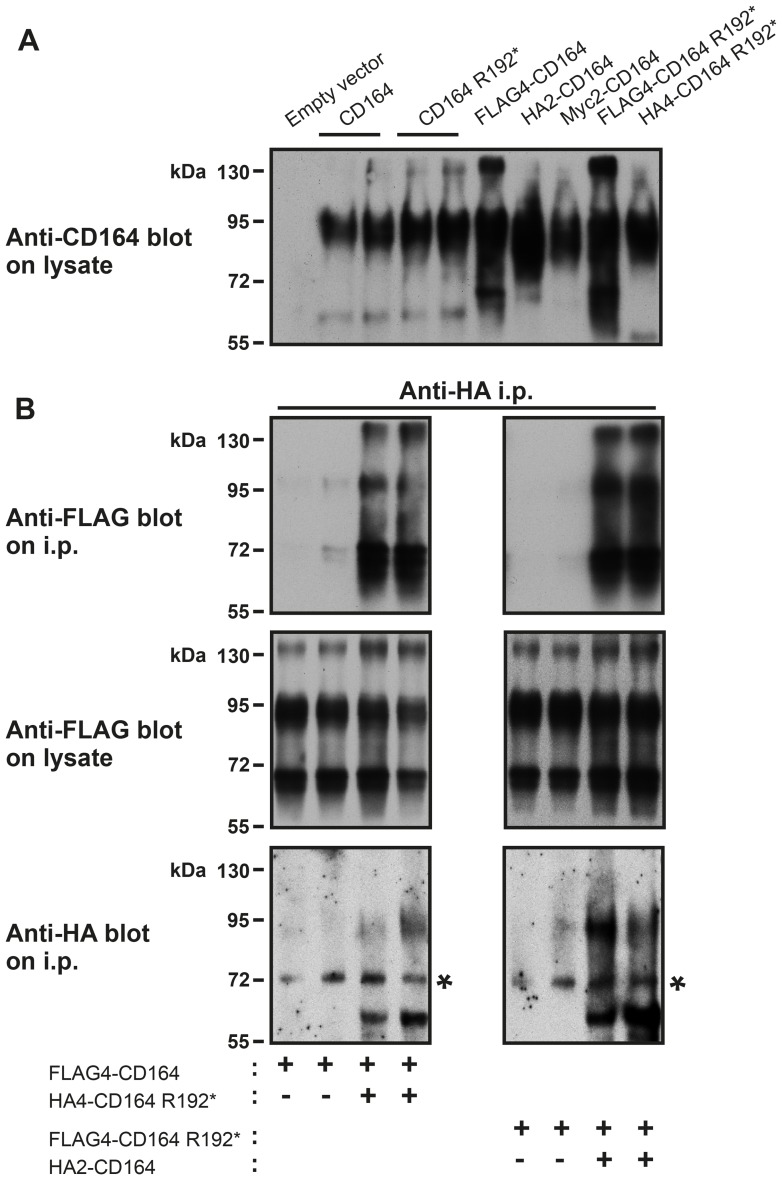
CD164 and CD164 R192* form heterodimers. HEK-293 cells were transiently transfected with empty vector, CD164 and CD164 R192*, either untagged or harboring various epitope tags and in various combinations. After 2 days, the cells were (A) lysed in SDS-PAGE sample buffer and analysed by immunoblotting using anti-human CD164 antibody or (B) lysed in immunoprecipitation buffer, whereafter CD164 or CD164 R192* were immunoprecipitated from the cell lysates using anti-HA antibody indicated. Aliquots of the immunoprecipitates or the pre-immunoprecipitation lysates were analysed by immunoblotting using the antibody indicated. SDS-PAGE was performed under reducing conditions. The asterisks indicate a non-specific band.

Because CD164 has been shown to form disulfide-linked homodimers [13,14], we speculated whether CD164 R192* could heterodimerize with CD164 WT. To this end, we generated expression constructs in which FLAG, HA or myc epitope tags were inserted at various positions in a relatively poorly conserved region immediately following the signal peptide of CD164. We first tested the expression of various tagged constructs compared to their untagged counter parts by transient transfection in HEK cells followed by immunoblotting analysis using antibody to human CD164. Untagged CD164 migrated as several bands with predominant species around 80–100 kDa under reducing conditions (Fig 5A). This is consistent with previous studies reporting migration of reduced CD164 as several bands ranging from 60–100 kDa depending on the cell line or tissue analysed. This migratory behavior is believed to be due to extensive and variable glycosylation of CD164 molecules [13–15]. We found that CD164 R192* expressed at similar or slightly higher levels and with identical molecular size as wild-type CD164, indicating that the mutation did not impair protein stability or glycosylation state. No signal was detected in empty vector transfected cells, showing that the endogenous CD164 was expressed at a low level compared to the exogenous CD164 in these experiments. The various epitope tags affected somewhat the CD164 expression level and the FLAG tag also the size distribution, with enhancement of species around 65 and 140 kDa, probably via effects on the glycosylation pattern. We next co-transfected HEK cells with distinctly tagged CD164 and CD164 R192* (or empty vector) in various combinations as indicated. Two days post-transfection, cells were lysed and wild-type or mutant CD164 immunoprecipitated using the appropriate anti-tag antibody, followed by immunoblotting for co-precipitation of the other CD164 form. This analysis showed that HA4-CD164 R192* was able to co-immunoprecipitate FLAG4-CD164 (Fig 5B left upper panel). Upon swapping of the tags, FLAG4-CD164 R192* was co-immunoprecipitated with HA2-CD164 (Fig 5B right upper panel). Control immunoblots demonstrated appropriate co-expression of the two constructs (Fig 5B middle and lower panel). Thus, in our experiments mutant CD164 was able to co-precipitate wild-type CD164 and vice versa demonstrating that mutant CD164 can form heterodimers with wild-type CD164 in HEK cells.

### No apparent effect of CD164 R192* on wild-type CD164 internalisation

**Fig 6:**
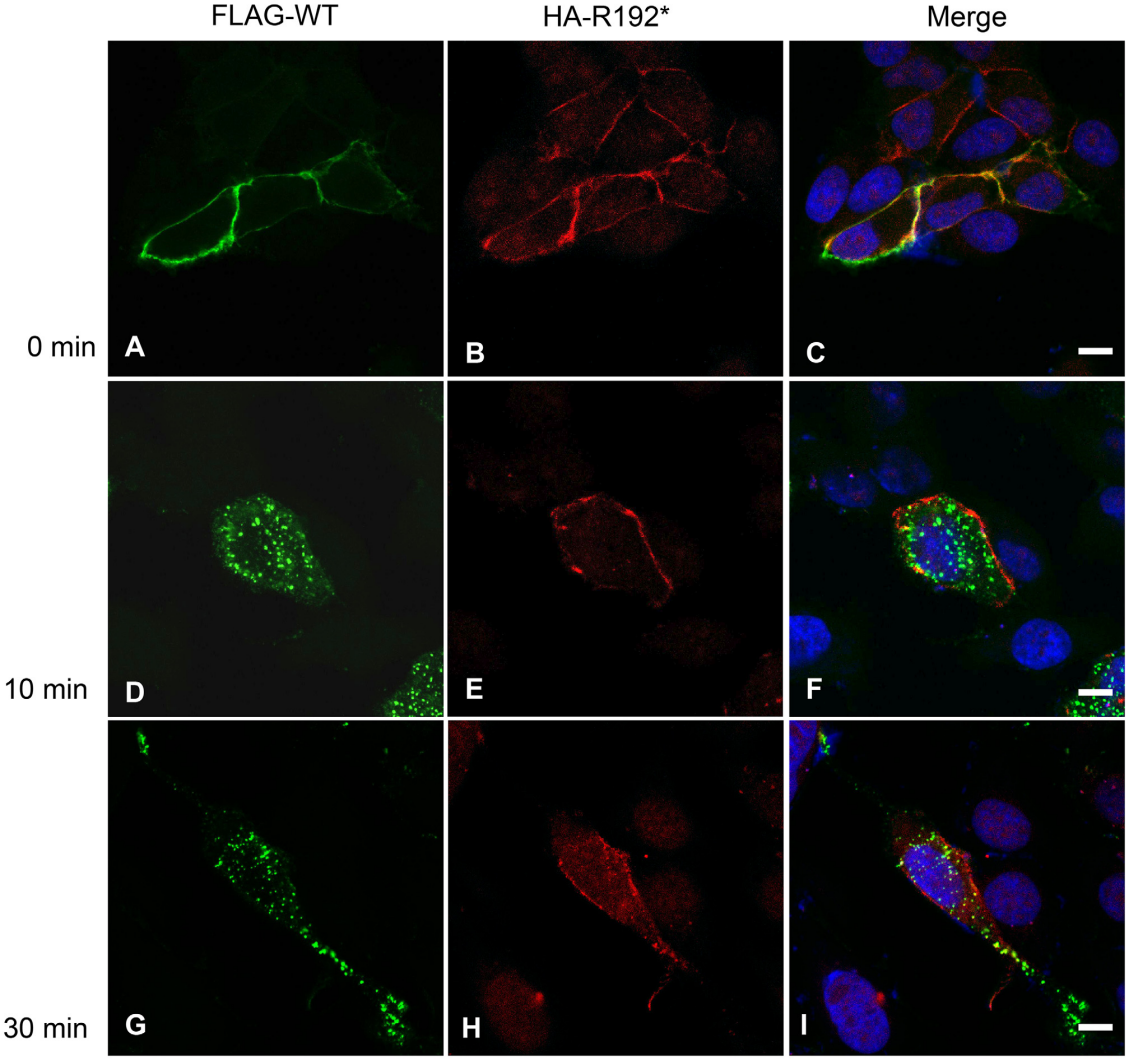
Internalization of full-length wild-type-FLAG and R192*-HA tagged CD164. Co-transfected HEK cells expressing wild-type CD164 tagged with the FLAG epitope (FLAG-CD164-WT) and truncated endolyn tagged with the HA epitope (HA-CD164-R192*) were seeded on cover slides. Following incubation with anti-FLAG and anti-HA antibodies on ice, the cells were either fixed (T0, 0 min) or incubated in complete medium without antibody for 10 (T10) and 30 (T30) minutes, respectively, and then fixed. FLAG-CD164-WT (A, D, G) and HA-CD164-R192* (B, E, H) was visualized using Alexa Fluor 488-labeled secondary antibody (green) and Alexa Fluor 568-labeled secondary antibody (red), respectively. Nuclear DNA was stained with DAPI (blue). Merged images are shown in (C, F, I). Imaging was performed on a confocal laser scanning microscope using 40×oil-immersion objective. Scale bar = 6 μm.

Given their ability to form heterodimers, we next tested if the internalization-deficient CD164 R192* mutant could negatively affect internalization of wild-type CD164. We co-transfected HEK cells with HA4-CD164 R192* and FLAG4-CD164 followed by double-staining of the cells with HA and FLAG antibodies at 0°C (Fig 6). Under these conditions of arrest of the endocytic machinery both wild-type and truncated CD164 was localized at the plasma membrane (Fig 6A–6C). However, after shifting the cells to internalization permitting conditions (37°C) most of the wild-type CD164 was internalized after 10 min with no further change at 30 min, whereas the majority of CD164 R192* maintained localisation on the plasma membrane (Fig 6D–6I). Thus, while these results support the findings on the internalization of wild-type and lack thereof for mutant CD164 presented in Fig 4, they do not support the idea that mutant CD164 R192* negatively affects internalization of wild-type CD164. It should be mentioned that in a minority of cells, we observed slow or no internalization of both wild-type and truncated CD164. Although we cannot completely rule out an effect of mutant CD164, we believe this observation is more likely explained by a non-functional internalization system in these cells.

### The CD164 c.574C>T mutant transcript survives nonsense-mediated mRNA decay

**Fig 7:**
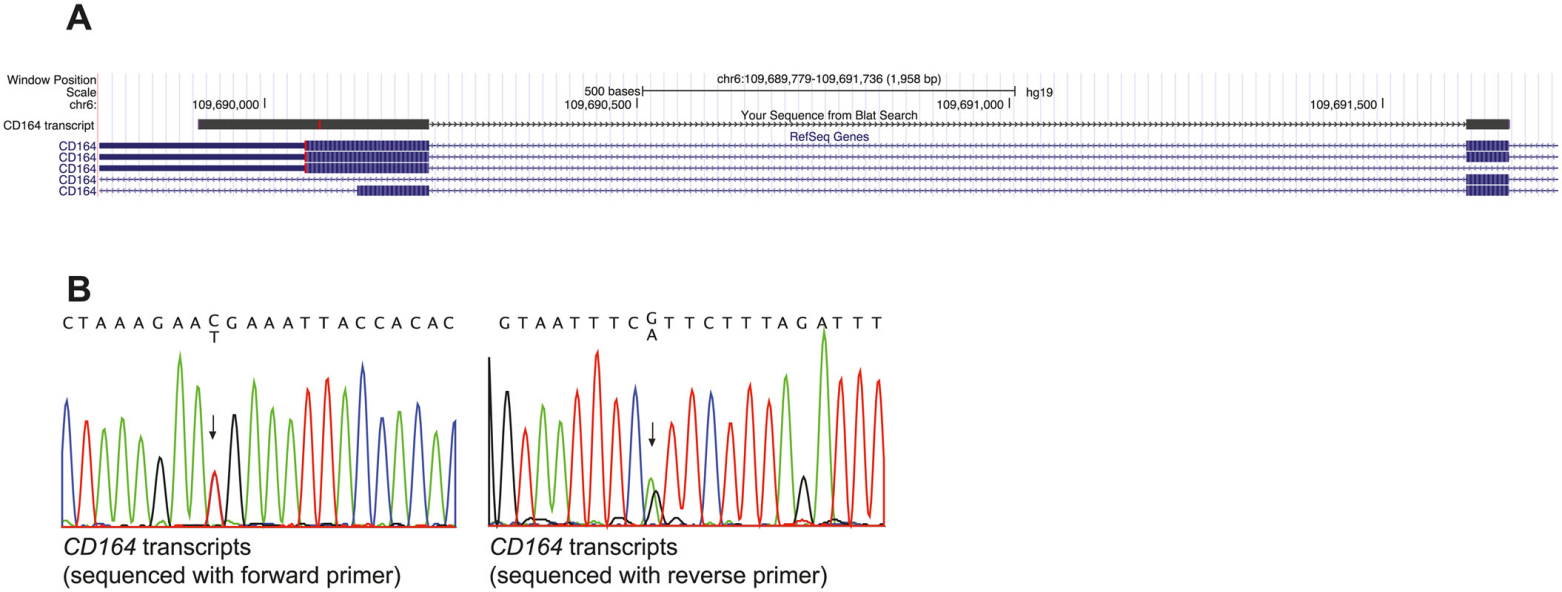
*CD164* transcripts with the c.574C>T (p.R192*) nonsense mutation escape NMD. (A) BLAT alignment of the sequenced cDNA (labeled “CD164 transcript” in the “your sequence from Blat search” track) from peripheral blood from a patient. The transcript does not contain intronic sequence indicating that the sequence is from mRNA. (B) Chromatogram of the c.574C>T mutation showed equal expression of both alleles in peripheral blood cells, demonstrating that the nonsense mutation is not degraded through NMD

Given the large effect of the p.R192* mutation on CD164 subcellular trafficking in our cell based assays, we speculated whether the transcript containing the mutation was expressed in cells from the affected family members. In mammalian cells, transcripts containing premature stop codons are generally degraded by nonsense-mediated mRNA decay (NMD). The efficiency of NDM, however, depends on the exact position of the premature stop codon [16]. We extracted RNA from a blood sample from the index patient (IV-5, Fig 1A) and after RT-PCR using intron spanning primers and Sanger sequencing, we aligned the obtained sequence to the human genome using BLAT to validate that it was from cDNA and not from genomic DNA (Fig 7A). We found that both the normal and mutated *CD164* transcripts were expressed in peripheral blood cells (Fig 7B), demonstrating that the CD164 c.574C>T transcript escapes NMD. This is consistent with the “55 bp rule” described for NMD, where the surveillance system in general seem to fail to distinguish premature stop codons if they are positioned in the last exon or in the second to last exon and located less than 55 bp from the final intron [17], which is the case for the present mutation.

### *CD164* is expressed in the cochlea of the rodent organ of Corti

**Fig 8:**
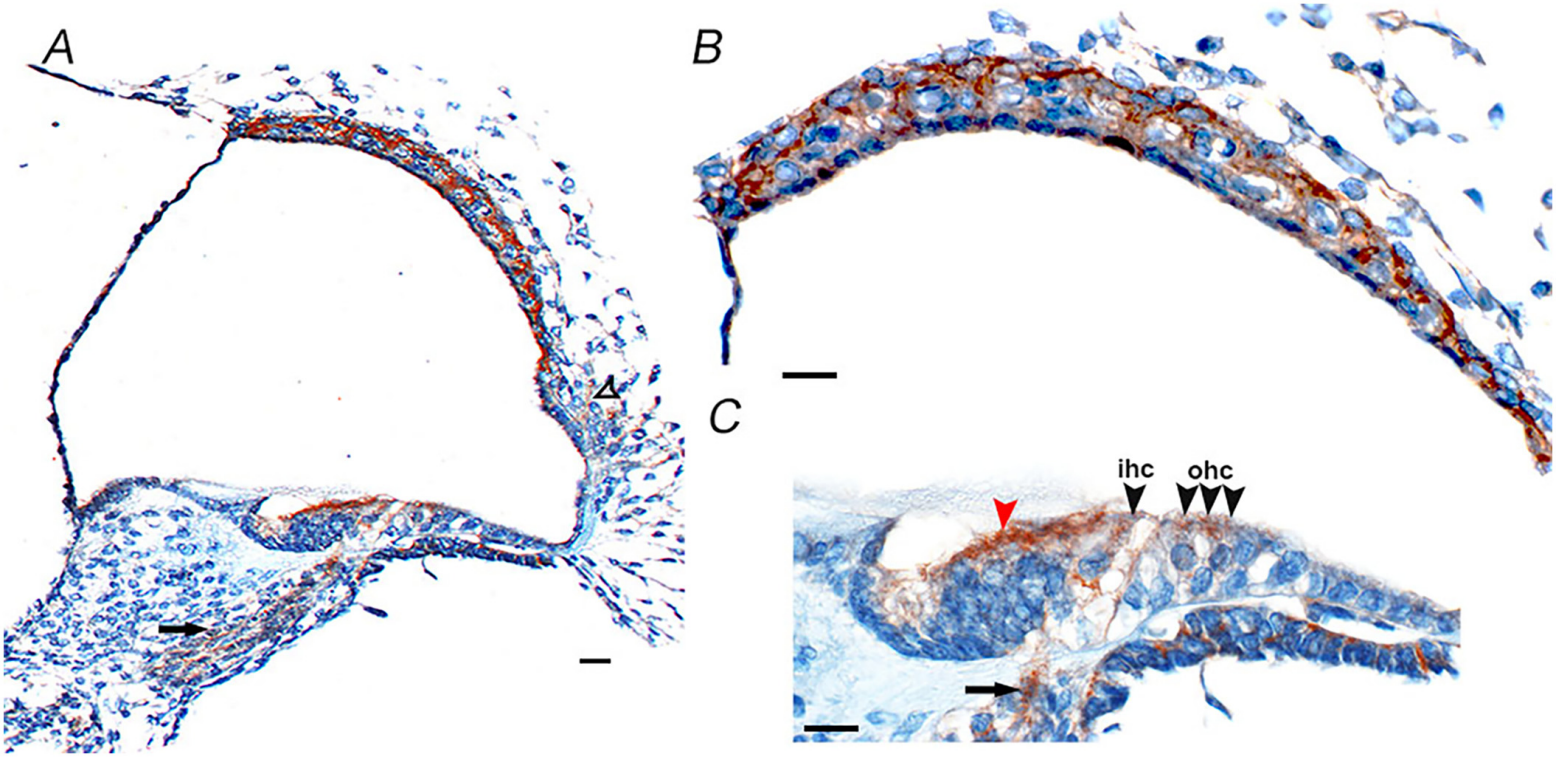
Cd164 expression in the mouse cochlea at postnatal day five. Immunohistochemistry shows cd164 expression (brown) in the cochlear neurons (arrows in A and C), inner hair cells (ihc) and outer hair cells (ohc) of the organ of Corti (black arrowheads in C), cells of Kolliker’s organ (red arrowhead in C), cells of the lateral wall behind the spiral prominence (open arrowhead in A) and in the stria vascularis (B). Scale bars: A: 10 μm, B, C: 5 μm.

For the gene to have a likely role in disease pathology, it should be expressed in the relevant tissue. From the publicly available BioGPS [18] database *CD164* transcripts appear to be widely expressed across different tissues in the human body, with high expression levels in the thyroid, whole blood, colon and small intestine, and medium expression in many other organs and lowest expression levels in the brain [18]. *CD164* transcripts are also expressed in the human fetal cochlea, according UniGene Hs. 520313, with inner ear data derived from Morton Human Fetal cDNA Library [19]. The detailed cellular distribution of CD164 at the protein level within the inner ear has however not been determined [20]. The protein expression pattern of cd164 in the inner ear was therefore investigated by staining of sections of mouse cochlea at postnatal day five using two different antibodies (Fig 8 and S5 Fig). This analysis indicated cd164 expression in the cochlear neurons, inner and outer hair cells of the organ of Corti, cells of Kolliker’s organ, cells in the lateral cochlear wall behind the spiral prominence and cells of the stria vascularis. The two antibodies showed the same expression pattern in the cochlea. The expression in the hair cells was weaker than in the other cell types, consistent with the mRNA expression pattern of cd164 in the Shared Harvard Inner-Ear Laboratory Database (SHIELD) database.

## Discussion

In this study, we mapped a novel locus (DFNA66) for NSHI to chromosome 6q15-21. The locus contained *FOXO3* and *SOBP*, known to cause deafness in mice, but Sanger sequencing and careful assessment did not identify any variation in these genes. By targeted sequence capture combined with NGS we instead identified a novel nonsense mutation in *CD164*, which was the only rare variant with a predicted functional impact, and thereby the best candidate in the region.

In our filtering strategy we did not filter solely on the presence in dbSNP, because with the increasing number of pathogenic variants being submitted to public databases, this may lead to low frequency causal variants being missed. Instead, we chose a conservative minor allele frequency threshold of 1%, which is a rather conservative threshold when performing mapping studies of high penetrant rare variants in Mendelian disorders [21].

In the family, the nonsense mutation segregated in all affected individuals, as well as to a 10-year old girl reported to be unaffected from multiple audiological examinations during her early childhood (Fig 1A, individual V-38). Interestingly, in a recent audiological follow-up after the finding of the mutation, a small dip in mid frequencies in her audiogram was found, which could be the first signs of an effect of the mutation, consistent with the broad range of age of onset observed in the family for the trait. If her hearing impairment progresses, all individuals carrying the variant will then display the phenotype, suggesting a high to complete penetrance with variable age of onset. Individual IV-21, who was not included in the linkage analysis due to recurrent severe otitis media in childhood, did not have the mutation. We therefore concluded that his hearing impairment is likely caused by the many ear infections.

In the search for rare disease causing mutations with high impact, linkage is an effective method for eliminating large fractions of the genome, but segregation and rarity alone is not sufficient to implicate a specific variant as pathogenic. In this study we therefore performed a number of functional studies to characterise CD164 and the effect of the mutation.

The YHTL motif, deleted by the c.574C>T nonsense mutation, is a canonical sorting motif known to be recognized by specific adaptor proteins in the cytosol, leading to subcellular trafficking of the transmembrane protein to endosomes and lysosomes [22]. In many transmembrane receptors (*e*.*g*. mannose 6-phosphate receptor and sortilin) the sorting motif mediates direct transport between the trans-Golgi network and endosomes, due to interaction with AP1 [22]. For other transmembrane proteins like CD164 and CD1 cellular trafficking to lysosomes also depend on AP3, but through different routes. Whereas newly synthesized CD1 seems to be captured by AP3 in the TGN for direct sorting to lysosomes, CD164's lower affinity for AP3, combined with a sorting signal residing in the luminal/extracellular domain, results in direct transport to the cell surface [23]. At the plasma membrane, the YHTL motif is recognized by AP2 and CD164 is subsequently rapidly endocytosed into early endosomes, a process known as the indirect route [10]. This is consistent with our functional data showing that CD164 R192* lacking the sorting motif is accumulated on the cell surface.

Such a dramatic effect on localisation of CD164 when perturbing the YHTL sorting motif has also been seen in other cell types, where point mutations of the critical tyrosine (Y) and leucine (L) residues in the YHTL motif were shown to lead to retention of CD164 at the plasma membrane [10,23,24]. We are the first to study the effect of an YHTL-disrupting CD164 mutation identified in humans. Taken together, the data suggest that abnormal trafficking of CD164 is consistently observed across different cell types when the YHTL sorting motif is perturbed.

The molecular mechanism through which truncated CD164 causes hearing loss is currently unknown. We have shown that the c.574C>T mutant transcript is not degraded by NMD in whole blood in patients, and that CD164 R192* is able to dimerize with wild-type CD164. We have also shown that CD164 R192* is trapped at the plasma membrane, but that the truncated protein does not appear to hold back wild-type CD164 on the surface in HEK cells, arguing against a direct dominant negative effect on wild-type CD164 internalization. However, it is possible that CD164 R192* may suppress other functions of wild-type CD164 via dimerization to cause hearing loss. It is also possible that the increased amounts of CD164 R192* protein at the plasma membrane could exert a “toxic” effect in cells in the inner ear. Other organ-specific diseases arising as a consequence of alterations in the sorting signals of individual plasma membrane proteins has been reviewed in [25,26].

CD164 has been shown to regulate CXCR4 signaling in hematopoietic precursor cells [27] and myoblasts [24]. However, none of the affected family members was evaluated for hematopoietic disorders.

Previously, CD164 have been studied in Drosophila and recently in zebrafish. In a study from 2006, Zhou et al. found that endolyn-deficient Drosophila mutants were arrested in embryonic and early larval development [28], and that a proportion of the growth-inhibited cells were undergoing apoptosis, suggesting a role for CD164 in cell proliferation. More recently, Mo et al., studied the kidney function in zebrafish embryos after morpholino knockdown of endolyn expression, and found that despite the pronephric kidney appeared morphologically normal, clearance of fluorescent dextran injected into the common cardinal vein was delayed, suggesting a defect in the regulation of water balance in the morphant embryos [29]. Interestingly, the authors found that the defects could be rescued by expression of rat endolyn, but not by expression of endolyn lacking the canonical YXXФ sorting motif, suggesting that correct kidney function require endolyn endocytosis at least in zebrafish [29]. In the present family there were no reports of renal disease. The creatinine and carbamide levels, measured in peripheral plasma in one of the affected family members, were found to be within normal range, and no microscopic kidney abnormalities were reported in an autopsy report of an affected family member, deceased in 2008. The phenotype of the morpholino zebrafish may however still be of some interest, since both the kidney and the inner ear contain polarised epithelial cells important for maintenance of fluid homeostasis. Furthermore, cd164 expression was detected in the stria vascularis (among other key functional sites) of the mouse cochlea, supporting the possibility of a role in endolymph homeostasis. As fluid homeostasis is important for correct hearing, this could be one possible mechanism through which CD164 is involved in hearing loss.

In conclusion we have identified a novel locus for hearing impairment with LOD score 5.1 and identified *CD164* as the most likely causative gene in the locus. Our data points towards an important role of CD164 in the function of the inner ear and suggest that the lack of the YXXФ motif, which is important for AP2 mediated endocytosis, underlies the hearing impairment in this family, however the exact molecular disease mechanism needs to be further investigated.

## Materials and Methods

The study was approved by the Danish Research Ethical Committee (reference numbers 20020036, KF 01–234/02 and KF 01–108/03), the medical ethics committee of the Radboud University Medical Center, the Institutional Review Boards of Quaid-i-Azam University and Baylor College of Medicine and Affiliated Hospitals. Informed consent was obtained from all family members who participated in the study.

Mouse studies were carried out in accordance with UK Home Office regulations and the UK Animals (Scientific Procedures) Act of 1986 (ASPA) under a UK Home Office licence, and the study was approved by the Wellcome Trust Sanger Institute’s Ethical Review Committee. Mice were culled using methods approved under this licence to minimize any possibility of suffering.

### Ascertainment of family

The proband was ascertained and the family pedigree constructed in collaboration between Department of Clinical Genetics, Vejle Hospital and Department of Audiology, Bispebjerg Hospital.

### Audiological examinations

One male (IV-5), with hearing impairment first diagnosed at about age 10 y, was examined several times. The audiograms at age 31 and at age 35 were similar, with 40 dB HL at 500 Hz, increasing to 70 dB HL at the frequencies 1000–4000 Hz, and improving to 20 dB HL at 8000 Hz (Fig 1B). His daughter (V-39) was diagnosed at neonatal hearing screening and carefully followed. She had at age 5 a sloping audiogram with 30–40 dB HL at frequencies 250–500 Hz, and 60–70 dB HL at 1000–2000 HZ and 50–60 dB HL at 4000–8000 Hz (S1 Fig). Between age 5 and 6, no progression was observed. A male in another branch of the family (V-24), experienced hearing impairment from the age of 3, and at age 6 an audiogram showed a basin shaped curve with 30dB HL at 500 HZ, dipping to 60 dB HL at 1000 HZ and 40 dB HL at 4000 HZ. At age 19, his audiogram showed 50 dB HL at 500 Hz, and a 60 dB HL at 1000–8000 HZ, thus illustrating progression (S1 Fig). Vestibular complaints were not reported subjectively. Individual IV-21 had symptoms of hearing impairment and numerous purulent childhood middle ear infections > 20 punctures of the eardrum, culminating with an operation for choleastoma, which is a known complication of middle ear infection. From his audiogram (S1 Fig) it was not possible unequivocally to determine if his hearing impairment was sensorineural or conductive (caused by the infections). His phenotype was considered unknown through the study.

### Genome-wide linkage analysis

Genomic DNA was extracted from peripheral blood samples. Ten affected and one unaffected individual (indicated in yellow in Fig 1A) were genotyped using the Human Mapping 50K SNP Xba240 Array (Affymetrix, High Wycombe, UK). Genotypes were called using the Genotyping Console (Affymetrix) and uploaded to the BCSNP data management platform (BC Platforms, Espoo, Finland). Data on a total of 58,958 markers was generated. Those markers with Mendelian errors, which were detected with MERLIN, were removed from the dataset (491 markers). Removal of monomorphic markers and LD pruning (using a sliding window of 50 SNPs and a r^2 threshold of 0.5) was performed using PLINK resulting in a filtered dataset of 11,034 markers in approximate linkage equilibrium with each other. MERLIN was also used to identify unlike genotypes, resulting in the removal of 221 genotypes from the dataset. Parametric linkage analysis was carried out with Merlin using an autosomal-dominant mode of inheritance with complete penetrance and a disease gene frequency of 0.0001, SNP allele frequencies from CEU and genetic distances from the Affymetrix 100K Marshfield cM map.

A follow-up analysis was performed by genotyping 26 available family members with seven microsatellite markers (D6S1595, D6S1644, D6S1613, D6S462, D6S416, D6S432, and D6S433) positioned within and just outside the linked region from the SNP analysis (S3 Table). Primer sequences were retrieved from the NCBI UniSTS database After PCR, the fragments were shipped to Eurofins Genomics (Ebersberg, Germany) for fragment analysis. Alleles were uploaded to BCSNP and parametric linkage analysis was performed with Mega2 [30] and SimWalk2 [31], which can handle large pedigrees. Allele frequencies were calculated from founders. Due to the variable age of onset of the hearing impairment in this family, the affection status of two apparently healthy children (16 years and 10 years old, respectively) was set to unknown. Similarly for one affected individual with multiple ear infections during childhood. Thus 23 individuals contributed to the follow-up linkage analysis. Disease allele frequency was set to 0.0001 and penetrance to 1.

### Sanger sequencing of eight candidate genes from the locus

All intron-exon boundaries and coding exons were sequenced for nine genes (*GJA10*, *POU3F2*, *C6orf168*, *LIN28B*, *Hsa-mir-587*, *SOBP*, *FOXO3 AMD1*, and *LAMA4)*. For *POU3F2*, we were able to PCR amplify, but not to Sanger sequence through a highly GC rich region (98% GCs) encoding a total of 21 glycine (Gly) residues in exon 1. Attempts to sequence this GC rich region (chr6:99,282,960–99,283,007) were performed by Sanger sequencing of two different PCR products, as well as providing the purified PCR product to Eurofins Genomics for direct Sanger sequencing using their custom service for difficult templates. As the same difficulty was found in two affected and two healthy control individuals, we assume that the failure is likely caused by polymerase failure and not by a mutation in the family. To exclude the presence of a trinucleotide expansion in this region, we amplified the region using a fluorescence-labeled primer pair followed by fragment length analysis at Eurofins Genomics. This analysis yielded a single peak for all samples analyzed (four affected, four control individuals), excluding that the sequencing failure across this region was caused by a trinucleotide expansion. Oligo sequences are listed in S3 Table.

### NimbleGen target-region capture and next-generation sequencing

A custom designed sequence capture array covering chr6:88,511,939–113,377,048 (hg19) was obtained from NimleGen (Roche NimbleGen, Madison, WI, USA). Genomic DNA from individual IV-31 (Fig 1A) was sheared by nebulization and universal adaptor oligonucleotides were ligated to the DNA. After this step, in order to enrich for the specific 6q region, the library was hybridized to the custom capture array. After washing to remove unhybridized material, captured molecules are recovered by heat-based elution and subjected to PCR amplification. The target-enriched library was quantified and subjected to deep sequencing on an Illumina Genome Analyzer, GAII using 36 bp reads. One lane of the flow cell was used for the sample.

The raw sequence reads were aligned to the reference genome (hg19, NCBI build 37) using Burrows-Wheeler Aligner (BWA) [32]. This generated a total of 3.8 Gb of sequence. In order to identify single nucleotide variants and indels Genome Analysis Toolkit (GATK) was used described in “Best Practice Variant Detection with the GATK v4” [33], which included removal of duplicate reads, local realignment around indels and base quality score recalibration before calling of genetic variants [34]. The sequencing depth and summary mapping statistics of the target region (S4 Table) were calculated using BEDTools [35], PICARD (http://picard.sourceforge.net), SamTools [36] and custom scripts. SNVs and indels were called using GATKs Unified genotyper [34] and subsequently SNVs were filtered in order to exclude SNVs with low mapping quality, low coverage and/or low quality scores. All variants passing this QC were indicated as PASS in the VCF file.

### Filtering in Ingenuity Variant Analysis

The VCF file was uploaded to Ingenuity Variant Analysis for variant filtering. The filtering steps were (1) kept PASS upstream pipeline filtering AND kept that are on chromosome 6 AND between positions 88556380 and 113518576, (2) excluded that are observed with an allele frequency greater than or equal to 1.0% of the genomes in the 1000 genomes project OR greater than or equal to 1.0% of the public Complete Genomics genomes OR greater than or equal to 1.0% of the NHLBI ESP exomes (All) (3) kept that are Frameshift, in-frame indel, or stop codon change OR Missense OR disrupt splice site upto 2.0 bases into intron OR structural variant (S2 Fig). We used Ingenuity Variant Analysis version 3.0.20140520 Content versions: Ingenuity Knowledge Base (Arrakis 140408.002), COSMIC (v68), dbSNP (Build 138 (08/09/2013)), 1000 Genome Frequency (v3), TargetScan (v6.2), EVS (ESP6500 0.0.21), JASPAR (10/12/2009), PhyloP hg18 (11/2009), PhyloP hg19 (01/2009), Vista Enhancer hg18 (10/27/2007), Vista Enhancer hg19 (12/26/2010), CGI Genomes (11/2011), SIFT (01/2013), BSIFT (01/2013), TCGA (09/05/2013), PolyPhen-2 (HumVar Training set 2011_12), Clinvar (02/11/2014).

### Genotyping of the *CD164* mutation

The *CD164* c.574C>T genotyping assays were developed by TIB MOLBIOL (Berlin, Germany) for the LightCycler 480 instrument (Roche, Hvidovre, Denmark). Oligo sequences are listed in S3 Table. Genotyping was performed on 26 members of the Danish family and 1200 Danish control individuals (500 medical students from Aarhus University and 700 anonymous Danish blood donors). No information on the hearing ability of the control individuals was available.

### Sequencing of *CD164* in individuals with unknown cause of hearing impairment

PCR primers were designed to amplify exons and surrounding intronic regions of the 7 exons of *CD164* (RefSeq nos. NM_006016.4 and NM_001142404.1). Primer sequences are available in S3 Table. PCR conditions are available upon request. In total 46 individuals were screened for *CD164* mutations. Among the tested individuals were the probands from five consanguineous Pakistani families with presumed recessive NSHL displaying linkage compatible with a locus on chromosome 6. These five hearing impaired probands were from families DEM4010 (LOD score 2.70), DEM4026 (LOD score 2.13), DEM4028 (LOD 1.23), DEM4059 (LOD score 3.00) and DEM4446B (LOD score 2.54).

### Plasmid constructions

#### Fusion proteins

To generate fluorescence constructs containing the C-terminal (CTR) of CD164, wild-type and CD164 R192* (with CD164 R192* lacking the last six residues RNYHTL), mCherry and eGFP were amplified by PCR and subcloned into pSECTAG2bzeo (Invitrogen) in reading frame with the ER signal peptide present in this vector. Overlapping oligodeoxynucleotides that contain the coding sequence for the transmembrane domain and C-terminal region (CTR) of CD164 wild-type as well as transmembrane domain and CD164 R192* truncated C-terminal domain of CD164, were annealed and filled up with deoxy-nucleotides before Eco*RI*/Xho*I* subcloning in pSECTAG2bzeo (Invitrogen). The resulting constructs were named pcSECTAG2bzeo-mCherry-CD164-WT-CTR, pcSECTAG2bzeo-eGFP-CD164-WT-CTR, SECTAG2bzeo-Cherry-CD164-R192*-CTR and SECTAG2bzeo-eGFP-CD164- R192*-CTR.

#### Intact proteins

To generate full-length CD164, wild-type and R192* CD164 cDNA was cloned into Zeo, Hyg or Neo versions of pcDNA3.1(+) (Invitrogen, Taastrup, Denmark) using the unique restriction sites Bam*HI* and Xba*I*, thereby forming the respective pcDNA3.1-CD164-WT-Zeo, pcDNA3.1-CD164-WT-Hyg and pcDNA3.1-CD164- R192*-Neo plasmids.

#### Tagged proteins

To generate epitope-tagged versions of CD164, the HA tag (YPYDVPDYA), triple FLAG tag (DYKDHDGDYKDHDIDYKDDDDK) or the myc tag (EQKLISEEDL) were inserted in a phylogenetically poorly conserved 34 amino acid region C-terminal to the signal peptide at the indicated positions: DKN(FLAG1)TTQ(HA2, myc2)HPNVTTLAPISNVTSA(FLAG3)PVTSLPLVTT(HA4, FLAG4)PA, with arbitrary numerals referring to the position of insertion. DNA encompassing this region and inserted tags were synthesized by Gene Oracle Inc (Mountain View, CA, USA) and cloned into CD164 or CD164 R192* in pcDNA3.1. Expression of the various CD164 variants was under transcriptional control of the cytomegalovirus (CMV) promoter. All constructs were verified by restriction analysis and sequencing.

### Transfection of cells

The human embryonic kidney cell line, HEK-293 (cat. no. CRL-1573, American Type Culture Collection, Boras, Sweden) was maintained and cultivated according to standard techniques [37]. Transiently transfected cells were obtained by means of X-tremeGENE 9 (Roche Applied Science, Hvidovre, Denmark) transfection experiments following the manufacturer’s instructions using 1.5 μg total plasmid DNA and 9 μl X-tremeGENE 9 transfection reagent. In brief, HEK cells were seeded in 35 mm glass bottom microwell dishes (MatTek, Ashland, MA, USA), and the next day they were co-transfected with pcDNA3.1-mCherry-CD164-WT-CTR and pcDNA3.1-eGFP-CD164-R192*-CTR. Stable transfected cells HEK cells were generated in T75 flasks using a total of 11.25 μg DNA (pcDNA3.1-CD164-WT-Zeo, pcDNA3.1-CD164-WT-Hyg or pcDNA3.1-CD164- R192*-Neo) and 33.75 μl X-tremeGENE 9 transfection reagent and selection of transfected cells were done using medium containing antibiotics (Zeocine 100 μg/ml (Invitrogen), Hygromycin 100 μg/ml (Invitrogen), or Neomycine (G418) 1.5 mg/ml (VWR, Herlev, Denmark)). Approximately one week after initiation of the selection procedure, non-transfected cells were dead and several positive clones were harvested after an additional week of the selection. Expression of CD164 was validated either by fluorescent microscopy of fluorescent marker genes (mCherry and eGFP), immunostaining of CD164 or by qPCR.

### Live imaging, immunostaining and internalization

For live imaging of CD164 fusion proteins, HEK cells were co-transfected in glass bottom 35mm dishes (MatTek) with pcDNA3.1-CD164-WT-CTR-mCherry and pcDNA3.1-CD164R192*-CTR-eGFP. Two days post transfection the medium was replaced with DMEM without phenol red and live pictures was captured on a confocal laser scanning microscope (LSM 780, Zeiss, Jena, Germany) using 63× water-immersion objective with a NA of 1.2. Immunostaining and internalization was performed essentially as previously described [38]. In brief, stable transfected HEK cells or HEK cells co-transfected with FLAG4-CD164-WT and HA4-CD164-R192* seeded on glass were incubated on ice for 10 min to stop the endocytic machinery and subsequently incubated on ice for 90 min in medium containing 5 μg/ml purified mouse anti-human CD164 antibodies (cat. no. 551296, BD Biosciences), or a mixture of monoclonal anti-FLAG M2 antibodies (cat. no. F3165, Sigma) and rabbit anti-HA antibodies (cat. no. H6908, Sigma). One fraction of the cells (designated T0) were fixed in 4% paraformaldehyde (Lillies buffer) (Buch & Holm, Herlev, Denmark) for 15 min at RT, and permeabilized with PBS containing 0.25% (w/v) Saponin (Sigma-Aldrich). The remaining cells were incubated further at 37°C in complete medium (without antibody) for 10 and 30 min, respectively. At the indicated time points cells were washed, fixed, and permeabilized as described above. Detection of CD164 in the stable transfected HEK cells was performed using secondary Alexa Fluor 488 goat anti-mouse antibody (1:400, cat. no. A11029, Invitrogen, Taastrup, Denmark). Detection of FLAG- and HA-tagged CD164 was obtained by using secondary Alexa Fluor 488 goat anti-mouse antibody (1:400, cat. no. A11029, Invitrogen) and Alexa Fluor 568 donkey anti-rabbit antibody (1:400, cat. no. A10042, Invitrogen), respectively. Nuclei were stained with 4´,6-Diamidino-2-phenylindole (Sigma-Aldrich) and mounted on SuperFrost glass slides (Hounisen, Risskov, Denmark). Sequential imaging was done on a confocal laser scanning microscope (LSM 780, Zeiss, Jena, Germany) using 40× oil-immersion objective with a NA of 1.3.

### Dimer formation analysis

HEK-293 cells in 35 mm plastic dishes were transiently transfected with untagged or epitope-tagged CD164 and CD164 R192* or empty pcDNA3.1 vector using X-tremeGENE 9, as described above, and cultured for 2 days. For CD164 protein expression analysis, cells were thereafter lysed in reducing SDS-PAGE sample buffer and subjected to immunoblotting using sheep anti-human CD164 primary antibody (AF5790) and horseradish peroxidase-coupled anti-sheep secondary antibody (HAF016), both from R&D Systems. For CD164 dimer formation analysis, cells were solubilized in immunoprecipitation buffer, as described [39]. Cell lysates were then incubated with 2 μg antibody to the HA tag (12CA5 clone) and immune complexes were precipitated using protein G agarose beads (16–266, Millipore). Aliquots of the immunoprecipitates or the pre-immunoprecipitation lysates were subjected to SDS-PAGE under reducing conditions followed by immunoblotting with horseradish peroxidase-coupled antibodies to the FLAG tag (Sigma-Aldrich A8592, M2 clone) or the HA tag. Secondary antibodies were detected by chemiluminescence (SuperSignal West Femto, #34095, Pierce).

### qPCR assay of cell lines

A qPCR assay to detect the ratio between wild-type and mutant transcripts as well as total expression of *CD164* in the double transfected cell lines was developed. Primers were designed to amplify total *CD164* transcripts (recognising both transcripts) as well as the mutated and wild-type transcript (allele specific primers). For each cell lines RNA was extracted from cell pellets using RNeasy (Qiagen) and cDNA was synthesized using iScript cDNA Synthesis kit (BIO-RAD) and 500 ng input RNA. Minus RT reactions were included to control for genomic DNA contamination. qPCR with was carried out for the transfected cell lines as well as untransfected HEK cells for control. The geometric mean of three genes (*ACTB*, *HTRP* and *TBP)* was used to normalize for cDNA content. All reactions were performed in triplicates. Fold changes were calculated relative to untransfected HEK cell. The relative amount of mutated and wild-type transcript within each cell line was calculated by taking the ratio of each transcript level to the level of total *CD164* transcripts.

### RT-PCR analysis of *CD164* transcript from an affected family member

Total RNA from peripheral blood lymphocytes was isolated from one of the affected family members (Fig 1A, IV-5) using the PAXgene Blood RNA System consisting of a blood collection tube (PAXgene Blood RNA Tube) and nucleic acid purification kit (PAXgene Blood RNA Kit) (Qiagen). The RNA was reverse-transcribed onto cDNA by using HT_11_V primers and the Superscript II kit (Invitrogen). RT-PCR was carried out with forward and revers primers positioned in exon 5 and 6 respectively, thereby spanning intron 5 (NM_006016.4) (S3 Table). The PCR product was sequenced on both strands using Sanger sequencing and aligned to the *CD164* gene using the BLAT program (BLAST like alignment tool).

### CD164 expression in mouse inner ear

Three wild-type mice at postnatal day five from the albino C57BL/6J-*Tyr* ^*c-Brd*^ inbred strain were used for the expression analysis. The heads of all samples were dissected in PBS before fixation for two days in 10% formalin at 4°C, washing, dehydrating and embedding in paraffin wax. Embedded samples were cut into 8μm thick sections along the sagittal plane. Immunohistochemistry was then carried out according to the manufacturer’s instructions on slides using the Ventana Discovery machine with the manufacturer’s reagents CC1 (cat.no 950–124), EZPrep (cat.no 950–100), LCS (cat.no 650–010), RiboWash (cat.no 760–105), Reaction Buffer (cat.no 95–300), and RiboCC (cat.no 760–107). The DABMap Kit (Ventana; cat.no 760–124) with hematoxylin counterstain (cat.no 760–2021) and bluing reagent (cat.no 760–2037) were used. All antibodies were diluted in ‘Antibody staining solution’: 10% fetal calf serum, 0.1% Triton, 2% BSA and 0.5% sodium azide in PBS. The primary antibodies used were anti-CD164 (SantaCruz, sc-33124, 1:75 and St.John’s Laboratory, STJ92095, 1:500). The secondary antibody used was Jackson ImmunoResearch biotin-conjugated donkey anti-rabbit (711-065-152, 1:100). The stained slides were examined and images obtained using an AxioCam HRc camera mounted on a Zeiss microscope.

### Web resources

The Hereditary Hearing loss Homepage (http://hereditaryhearingloss.org)

OMIM—Online Mendelian Inheritance in Man (www.omim.org/)

PICARD (http://picard.sourceforge.net)

SMART database (smart.embl-heidelberg.de)

NetOGlyc 4.0 Server (http://www.cbs.dtu.dk/services/NetOGlyc/)

dbSNP (http://www.ncbi.nlm.nih.gov/SNP/)

1000 Genomes project (http://www.1000genomes.org)

Exome Variant Server database (http://evs.gs.washington.edu/EVS/)

UCSC Genome Browser (http://genome.ucsc.edu)

BioGPS (http://biogps.org)

GTEx (http://www.gtexportal.org/home/)

Morton Human Fetal Cochlea cDNA Library EST Data **(** http://brighamandwomens.org/Research/labs/BWH_Hearing/Cochlear_ESTs.aspx)

SHIELD: Shared Harvard Inner-Ear Laboratory Database (https://shield.hms.harvard.edu)

## Supporting Information

S1 Fig Audiograms from six individuals in the large Danish family with NSHI. Audiograms are from individual IV-22, IV-31, IV-21, V-23, V-39, and V-24.
(TIF)

S2 Fig Variant analysis and filtering. An algorithm in Ingenuity Variant Analysis was used for filtering all variants identified in the locus from the custom capture array, with numbers of variants left after each filtering step indicated.
(TIF)

S3 Fig Chromatogram of the region containing rs11542733. Sanger sequencing was not able to verify the presence of rs11542733 in CEPH 1341–13.
(TIF)

S4 Fig Quantitative PCR assay. A. The ratio of wild-type and mutant CD164 transcript for each cell line. B. The total CD164 expression indicated as foldchange compared to untransfected HEK cells. The average of three housekeeping genes was used for normalization. WT: HEK cell line transfected with wild-type construct. MUT: HEK cell line transfected with mutant transcript.
(TIF)

S5 Fig Cd164 expression in the mouse cochlea at postnatal day five performed with a different antibody (St. John’s Laboratory). Cd164 expression was confirmed in the spiral ganglions neurons, hair cells in the organ of Corti, cells of Kolliker’s organ, cells of the spiral prominence and in the stria vascularis. This antibody also shows cd164 expression in Claudius cells. Scale bar; 10 μm.
(TIFF)

S1 Table Parametric LOD score on chromosome 6 from the initial SNP-based genome-wide linkage analysis including 11 individuals. (DOCX)

S2 Table Genes within the locus identified from the SNP analysis. The region contain *SOBP* and *FOXO3*, which are genes involved in deafness in the mouse, but Sanger sequencing and careful check of all coding exons did not identify any variation in these genes.
(DOCX)

S3 Table Oligo and primer sequences used in this study. (DOCX)

S4 Table Statistics on target sequence capture array and NGS of the DNA sample from individual IV-31. (DOCX)
