# Supplementary figures and images for: A Novel Locus Harbouring a Functional CD164 Nonsense Mutation Identified in a Large Danish Family with Nonsyndromic Hearing Impairment
Source: PLoS Genet. 2015 Jul 21;11(7):e1005386. doi: 10.1371/journal.pgen.1005386 (PMC4510537; doi:10.1371/journal.pgen.1005386)

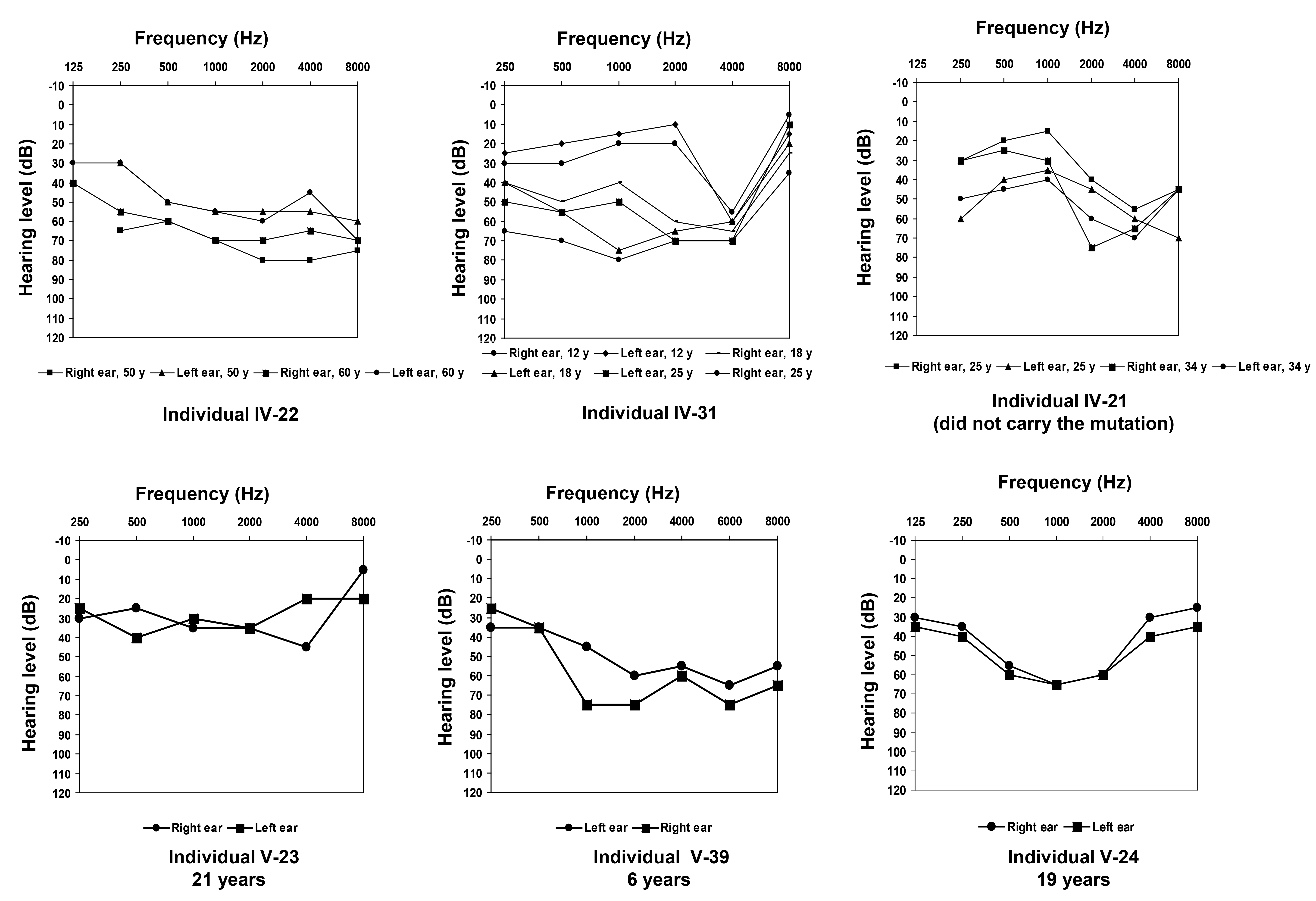

Supplement: S1 Fig — Audiograms are from individual IV-22, IV-31, IV-21, V-23, V-39, and V-24. (TIF) [file pgen.1005386.s001.tif]

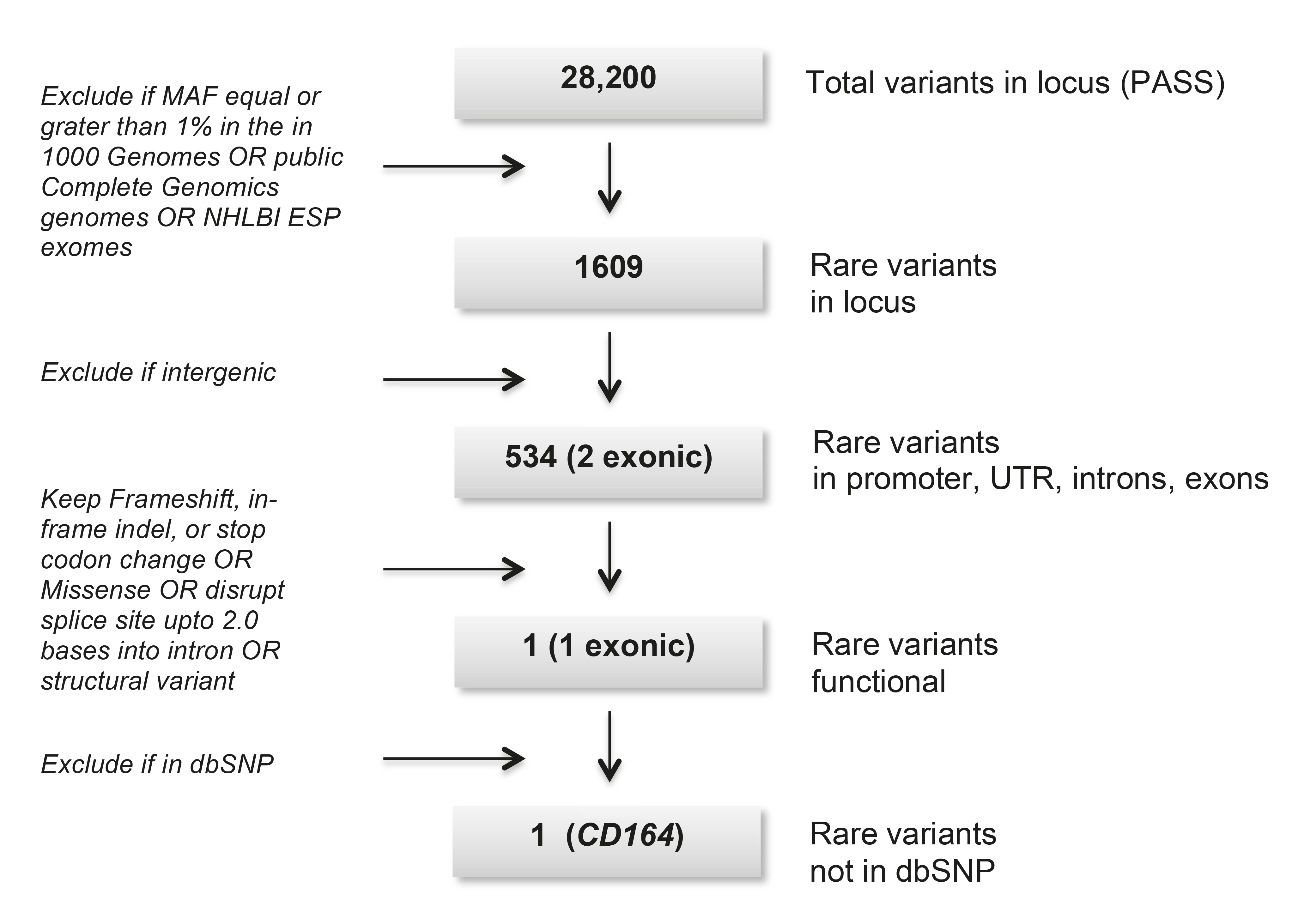

Supplement: S2 Fig — An algorithm in Ingenuity Variant Analysis was used for filtering all variants identified in the locus from the custom capture array, with numbers of variants left after each filtering step indicated. (TIF) [file pgen.1005386.s002.tif]

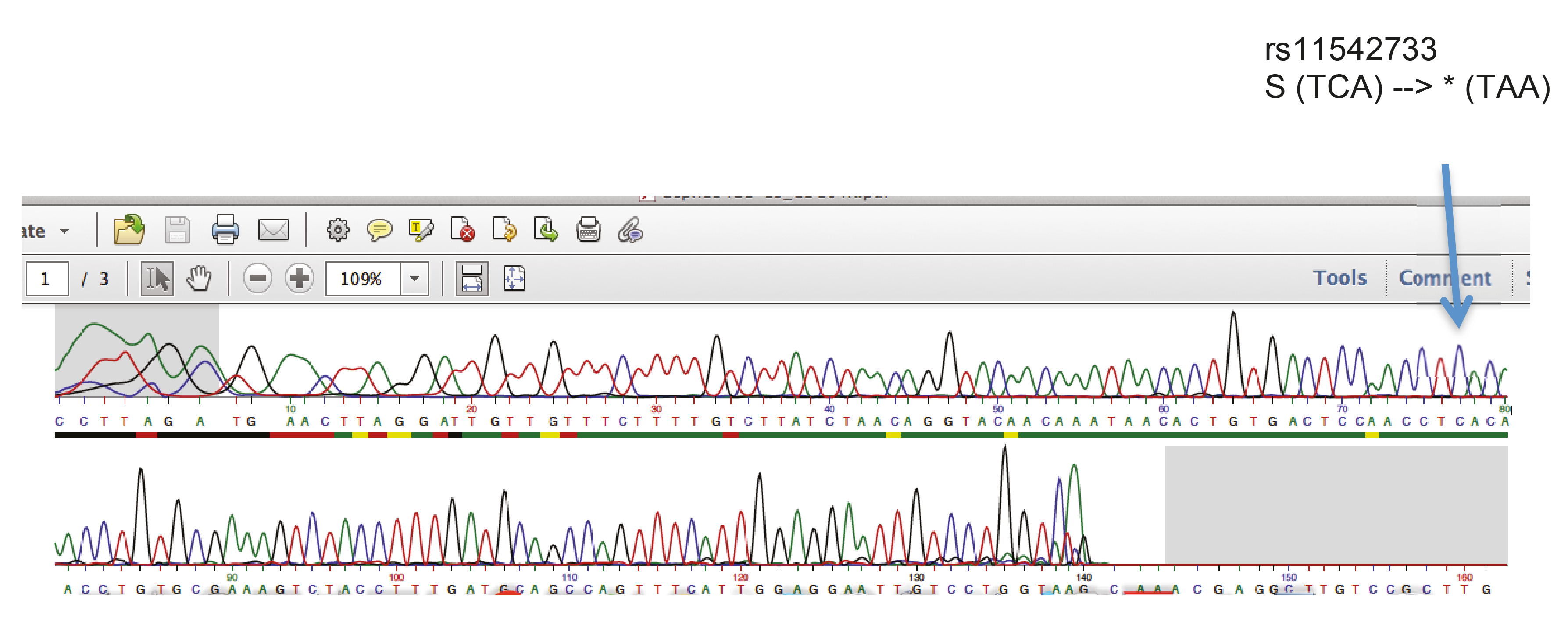

Supplement: S3 Fig — Sanger sequencing was not able to verify the presence of rs11542733 in CEPH 1341–13. (TIF) [file pgen.1005386.s003.tif]

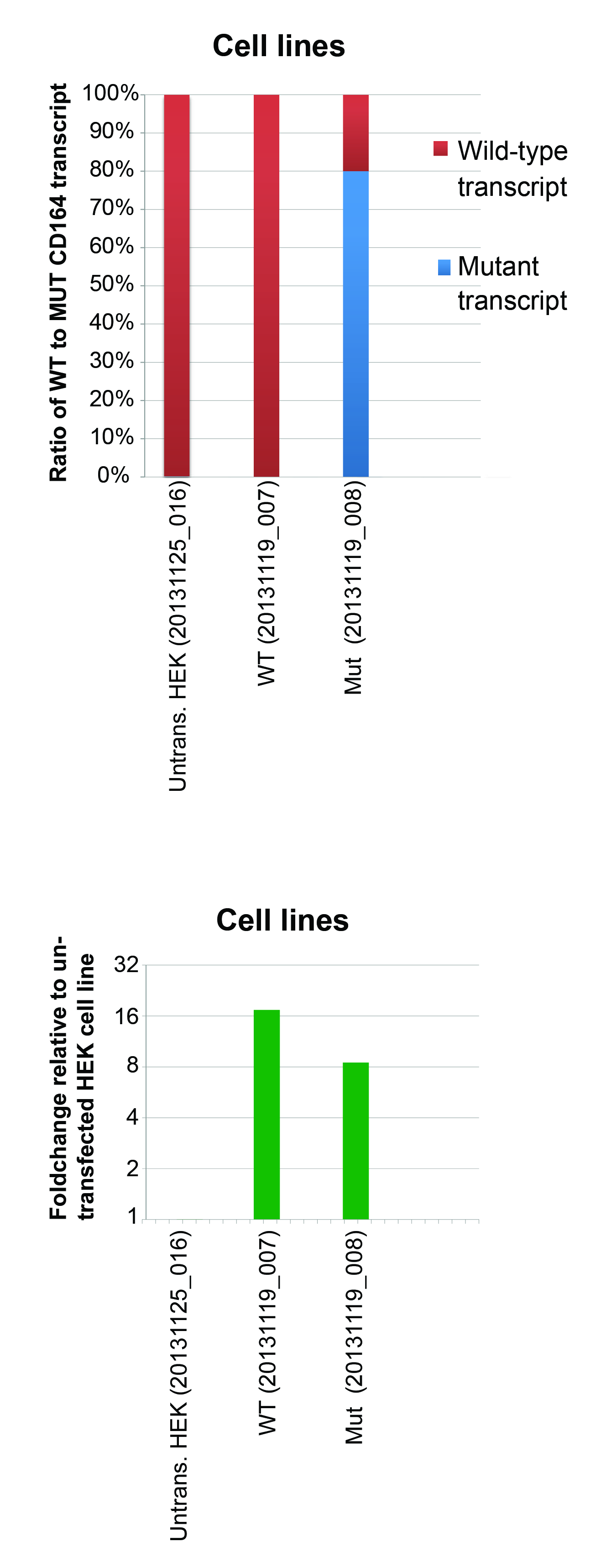

Supplement: S4 Fig — A. The ratio of wild-type and mutant CD164 transcript for each cell line. B. The total CD164 expression indicated as foldchange compared to untransfected HEK cells. The average of three housekeeping genes was used for normalization. WT: HEK cell line transfected with wild-type construct. MUT: HEK cell line transfected with mutant transcript. (TIF) [file pgen.1005386.s004.tif]

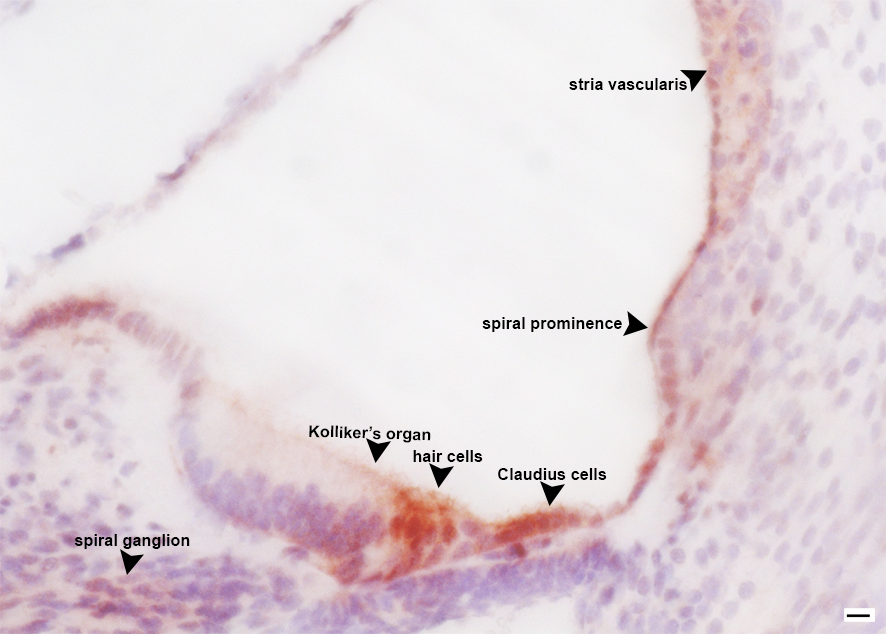

Supplement: S5 Fig — Cd164 expression was confirmed in the spiral ganglions neurons, hair cells in the organ of Corti, cells of Kolliker’s organ, cells of the spiral prominence and in the stria vascularis. This antibody also shows cd164 expression in Claudius cells. Scale bar; 10 μm. (TIFF) [file pgen.1005386.s005.tiff]
