# Supplementary material for: A Novel Locus Harbouring a Functional CD164 Nonsense Mutation Identified in a Large Danish Family with Nonsyndromic Hearing Impairment
Source: PLoS Genet. 2015 Jul 21;11(7):e1005386. doi: 10.1371/journal.pgen.1005386 (PMC4510537; doi:10.1371/journal.pgen.1005386)
Supplement: S1 Table — (DOCX) [file pgen.1005386.s006.docx]

| **Marker** | **LOD** | **Comment** |
| --- | --- | --- |
| RS1074688 | -0.088 |  |
| RS7750840 | -0.088 |  |
| RS950030 | -0.088 |  |
| RS1891754 | -0.088 |  |
| RS1588201 | -0.088 |  |
| RS952571 | -0.088 |  |
| RS4099964 | -0.088 |  |
| RS2268994 | -0.088 |  |
| RS9294390 | -0.088 | First proximal SNP outside region from SNP based linkage |
| RS648549 | 2.802 |  |
| RS366676 | 3.117 |  |
| RS10485169 | 3.603 |  |
| RS2875546 | 3.611 |  |
| RS2325098 | 3.611 |  |
| RS1923416 | 3.609 |  |
| RS2207553 | 3.609 |  |
| RS764108 | 3.609 |  |
| RS4707614 | 3.610 |  |
| RS1353227 | 3.610 |  |
| RS157692 | 3.611 |  |
| RS1923064 | 3.611 |  |
| RS10485409 | 3.611 |  |
| RS10485408 | 3.611 |  |
| RS10485328 | 3.610 |  |
| RS9294506 | 3.610 |  |
| RS369910 | 3.611 |  |
| RS9294511 | 3.611 |  |
| RS2325398 | 3.611 |  |
| RS1493554 | 3.611 |  |
| RS7739625 | 3.611 |  |
| RS611748 | 3.611 |  |
| RS1365709 | 3.611 |  |
| RS958847 | 3.611 |  |
| RS1824423 | 3.611 |  |
| RS1590384 | 3.611 |  |
| RS1930931 | 3.611 |  |
| RS1343850 | 3.611 |  |
| RS10485026 | 3.611 |  |
| RS474690 | 3.611 |  |
| RS574793 | 3.611 |  |
| RS2021314 | 3.611 |  |
| RS452854 | 3.611 |  |
| RS62827 | 3.611 |  |
| RS10485022 | 3.611 |  |
| RS2502004 | 3.611 |  |
| RS794672 | 3.610 |  |
| RS197892 | 3.610 |  |
| RS2000311 | 3.611 |  |
| RS2147716 | 3.611 |  |
| RS4515397 | 3.611 |  |
| RS1972474 | 3.611 |  |
| RS1359932 | 3.611 |  |
| RS2179537 | 3.611 |  |
| RS10485379 | 3.611 |  |
| RS946120 | 3.611 |  |
| RS1338551 | 3.611 |  |
| RS10484867 | 3.611 |  |
| RS150396 | 3.610 |  |
| RS2067012 | 3.610 |  |
| RS2029964 | 3.611 |  |
| RS6912685 | 3.611 |  |
| RS10499025 | 3.611 |  |
| RS924974 | 3.611 |  |
| RS9322033 | 3.611 |  |
| RS705607 | 3.610 |  |
| RS4840159 | 3.610 |  |
| RS10485300 | 3.610 |  |
| RS873649 | 3.610 |  |
| RS7760434 | 3.610 |  |
| RS2255496 | 3.611 |  |
| RS2399711 | 3.610 |  |
| RS10485275 | 3.610 |  |
| RS9322655 | 3.610 |  |
| RS1934009 | 3.610 |  |
| RS1991665 | 3.610 |  |
| RS473259 | 3.610 |  |
| RS155510 | 3.610 |  |
| RS1341112 | 3.610 |  |
| RS4132193 | 3.610 |  |
| RS7759427 | 3.610 |  |
| RS722366 | 3.610 |  |
| RS952063 | 3.610 |  |
| RS7748283 | 3.610 |  |
| RS2743562 | 3.610 |  |
| RS3844148 | 3.610 |  |
| RS803520 | 3.610 |  |
| RS9320161 | 3.610 |  |
| RS898895 | 3.610 |  |
| RS1026619 | 3.611 |  |
| RS6940205 | 3.610 |  |
| RS1884270 | 3.610 |  |
| RS319119 | 3.610 |  |
| RS7745587 | 3.610 |  |
| RS928222 | 3.603 |  |
| RS531111 | 3.603 |  |
| RS4945820 | 3.603 |  |
| RS528917 | 3.603 |  |
| RS10484253 | 3.603 |  |
| RS352849 | 3.603 |  |
| RS351736 | 3.603 |  |
| RS10499053 | 3.603 |  |
| RS2025149 | 3.603 |  |
| RS4307211 | 3.603 |  |
| RS1040903 | 3.604 |  |
| RS2297939 | 3.604 |  |
| RS1989574 | 3.604 |  |
| RS2072020 | 3.605 |  |
| RS7766236 | 3.605 |  |
| RS1935691 | 3.605 |  |
| RS10499066 | 2.627 |  |
| RS2881622 | 2.334 |  |
| RS6910441 | -0.390 | First distal SNP outside region from SNP based linkage |
| RS9320435 | -0.389 |  |
| RS1388219 | -0.390 |  |
| RS10484461 | -0.387 |  |
| RS9320460 | -0.386 |  |
| RS9285411 | -0.386 |  |
| RS3862822 | -0.386 |  |
| RS10499076 | -0.389 |  |
