# Supplementary material for: A Novel Locus Harbouring a Functional CD164 Nonsense Mutation Identified in a Large Danish Family with Nonsyndromic Hearing Impairment
Source: PLoS Genet. 2015 Jul 21;11(7):e1005386. doi: 10.1371/journal.pgen.1005386 (PMC4510537; doi:10.1371/journal.pgen.1005386)
Supplement: S2 Table — The region contain SOBP and FOXO3, which are genes involved in deafness in the mouse, but Sanger sequencing and careful check of all coding exons did not identify any variation in these genes. (DOCX) [file pgen.1005386.s007.docx]

| **Gene symbol** | **UCSC Description** |
| --- | --- |
| *CNR1* | cannabinoid receptor 1 (brain) |
| *RNGTT* | RNA guanylyltransferase and 5'-phosphatase |
| *PNRC1* | proline-rich nuclear receptor coactivator 1 |
| *SRSF12* | serine/arginine-rich splicing factor 12 |
| *PM20D2* | peptidase M20 domain containing 2 |
| *GABRR1* | gamma-aminobutyric acid (GABA) receptor, rho 1 |
| *GABRR2* | gamma-aminobutyric acid (GABA) receptor, rho 2 |
| *UBE2J1* | ubiquitin-conjugating enzyme E2, J1, U |
| *RRAGD* | Ras-related GTP binding D |
| *ANKRD6* | ankyrin repeat domain 6 |
| *LYRM2* | LYR motif containing 2 (LYRM2), non-coding RNA |
| *MDN1* | MDN1, midasin homolog (yeast) |
| *CASP8AP2* | caspase 8 associated protein 2 |
| *GJA10* | gap junction protein, alpha 10 |
| *BACH2* | BTB and CNC homology 1, basic leucine zipper transcription factor 2 |
| *MIR4464* | microRNA 4464 |
| *MAP3K7* | mitogen-activated protein kinase kinase kinase 7 |
| *MIR4643* | microRNA 4643 |
| *EPHA7* | EPH receptor A7 |
| *TSG1* | tumor suppressor TSG1, non-coding RNA. |
| *MANEA* | mannosidase, endo-alpha |
| *FUT9* | fucosyltransferase 9 (alpha (1,3) fucosyltransferase) |
| *UFL1* | UFM1-specific ligase 1 |
| *FHL5* | four and a half LIM domains 5 |
| *GPR63* | G protein-coupled receptor 63 |
| *NDUFAF4* | NADH dehydrogenase (ubiquinone) 1 alpha subcomplex, assembly factor 4, nuclear gene encoding mitochondrial protein |
| *KLHL32* | kelch-like 32 (Drosophila) |
| *MIR548H3* | microRNA 548h-3 |
| *MMS22L* | MMS22-like, DNA repair protein |
| *MIR2113* | microRNA 2113 |
| *POU3F2* | POU class 3 homeobox 2 |
| *FBXL4* | F-box and leucine-rich repeat protein 4 |
| *C6orf168* | chromosome 6 open reading frame 168 |
| *COQ3* | coenzyme Q3 homolog, methyltransferase (S. cerevisiae) |
| *PNISR* | PNN-interacting serine/arginine-rich protein |
| *USP45* | ubiquitin specific peptidase 45 |
| *LOC100130890* | uncharacterized LOC100130890 |
| *CCNC* | cyclin C (CCNC) |
| *PRDM13* | PR domain containing 13 |
| *MCHR2* | melanin-concentrating hormone receptor 2 |
| *SIM1* | single-minded homolog 1 (Drosophila) |
| *ASCC3* | activating signal cointegrator 1 complex subunit 3 |
| *GRIK2* | glutamate receptor, ionotropic, kainate 2 |
| *HACE1* | HECT domain and ankyrin repeat containing, E3 ubiquitin protein ligase 1 |
| *LIN28B* | lin-28 homolog B (C. elegans) |
| *BVES* | blood vessel epicardial substance |
| *C6orf112* | chromosome 6 open reading frame 112, non-coding RNA. |
| *POPDC3* | popeye domain containing 3 |
| *PREP* | prolyl endopeptidase |
| *PRDM1* | PR domain containing 1, with ZNF domain |
| *ATG5* | ATG5 autophagy related 5 homolog (S. cerevisiae) |
| *AIM1* | absent in melanoma 1 |
| *RTN4IP1* | reticulon 4 interacting protein 1 (RTN4IP1), nuclear gene encoding mitochondrial protein |
| *QRSL1* | glutaminyl-tRNA synthase (glutamine-hydrolyzing)-like 1 |
| *C6orf203* | chromosome 6 open reading frame 203 |
| *BEND3* | BEN domain containing 3 |
| *PDSS2* | prenyl (decaprenyl) diphosphate synthase, subunit 2 |
| *SOBP* | sine oculis binding protein homolog (Drosophila) |
| *SCML4* | sex comb on midleg-like 4 (Drosophila) |
| *SEC63* | SEC63 homolog (S. cerevisiae) |
| *OSTM1* | osteopetrosis associated transmembrane protein 1 |
| *NR2E1* | nuclear receptor subfamily 2, group E, member 1 |
| *SNX3* | sorting nexin 3 |
| *LACE1* | lactation elevated 1 |
| *FOXO3* | forkhead box O3 |
| *ARMC2* | armadillo repeat containing 2 |
| *SESN1* | sestrin 1 |
| *CEP57L1* | centrosomal protein 57kDa-like 1 |
| *AK094715* | RecName: Full=Transmembrane protein FLJ37396 |
| *CCDC162P* | coiled-coil domain containing 162, pseudogene, non-coding RNA. |
| *C6orf185* | chromosome 6 open reading frame 185, mRNA (cDNA clone IMAGE:40112263), partial cds. |
| *CD164* | CD164 molecule, sialomucin |
| *PPIL6* | peptidylprolyl isomerase (cyclophilin)-like 6 |
| *SMPD2* | sphingomyelin phosphodiesterase 2, neutral membrane (neutral sphingomyelinase) |
| *MICAL1* | microtubule associated monoxygenase, calponin and LIM domain containing 1 |
| *ZBTB24* | zinc finger and BTB domain containing 24 |
| *AKD1* | adenylate kinase domain containing 1 |
| *FIG4* | FIG4 homolog, SAC1 lipid phosphatase domain containing (S. Cerevisiae) |
| *GPR6* | G protein-coupled receptor 6 |
| *WASF1* | WAS protein family, member 1 |
| *CDC40* | cell division cycle 40 homolog (S. cerevisiae) |
| *C6orf186* | chromosome 6 open reading frame 186 |
| *DDO* | D-aspartate oxidase |
| *SLC22A16* | solute carrier family 22 (organic cation/carnitine transporter), member 16 |
| *CDK19* | cyclin-dependent kinase 19 |
| *BC047513* | cell division cycle 2-like 6 (CDK8-like), mRNA (cDNA clone IMAGE:5296862) |
| *AMD1* | adenosylmethionine decarboxylase 1 |
| *GTF3C6* | general transcription factor IIIC, polypeptide 6, alpha 35kDa |
| *RPF2* | ribosome production factor 2 homolog (S. cerevisiae) |
| *GSTM2P1* | glutathione S-transferase mu 2 (muscle) pseudogene 1, non-coding RNA. |
| *SLC16A10* | solute carrier family 16, member 10 (aromatic amino acid transporter) |
| *KIAA1919* | KIAA1919 |
| *REV3L* | REV3-like, catalytic subunit of DNA polymerase zeta (yeast) |
| *TRAF3IP2-AS1* | TRAF3IP2 antisense RNA 1 (non-protein coding) |
| *TRAF3IP2* | TRAF3 interacting protein 2 |
| *FYN* | FYN oncogene related to SRC, FGR, YES |
| *WISP3* | WNT1 inducible signaling pathway protein 3 |
| *TUBE1* | tubulin, epsilon 1 |
| *C6orf225* | chromosome 6 open reading frame 225 |
| *LAMA4* | laminin, alpha 4 |
| *RFPL4B* | ret finger protein-like 4B |
