# Supplementary material for: A Novel Locus Harbouring a Functional CD164 Nonsense Mutation Identified in a Large Danish Family with Nonsyndromic Hearing Impairment
Source: PLoS Genet. 2015 Jul 21;11(7):e1005386. doi: 10.1371/journal.pgen.1005386 (PMC4510537; doi:10.1371/journal.pgen.1005386)
Supplement: S3 Table — (DOCX) [file pgen.1005386.s008.docx]

| **Sequencing CEPH individual 1341.13** | **Sequence (5´- 3´orientation)** |  |
| --- | --- | --- |
| CD164rs11542733f | GACCAGGACAATTCCTCCAA |  |
| CD164rs11542733r | GAGGCTCAGGTGCATCTTTA |  |
|  |  |  |
| **Microsatellite markers** | **Genetic position (Marshfield cM)** | **Genomic position hg19 (Mb)** |
| D6S1595 | 92.85 | 88.5 |
| D6S1644 | 96.05 | 89.7 |
| D6S1613 | 97.11 | 90.6 |
| D6S462 | 99.01 | 90.9 |
| D6S416 | 118.64 | 112.5 |
| D6S432 | 119.47 | 112.9 |
| D6S433 | 121.97 | 118.6 |
|  |  |  |
| **Sequencing primers (Aarhus)** | **Sequence (5´- 3´orientation)** | **Amplicon length** |
| CD164Ex1-f | TTGCGAGCCTTAGCTTTCTC |  |
| CD164Ex1-r | TCGACTTGCAACACTTCGAG | 481 bp |
| CD164Ex2-f | CATTGATTTCATGCATCTCTCC |  |
| CD164Ex2-r | GCAACTGGCACTTTAAAGGAGT | 266 bp |
| CD164Ex3-f | AGGAGAATGACAGGGTTTGGT |  |
| CD164Ex3-r | GCACTGAAACAAGGCTTTCTG | 331 bp |
| CD164Ex4-f | GGAGGAAGGGAGGTGTTGATA |  |
| CD164Ex4-r | CAGCAAATCTGCTCACATTCA | 476 bp |
| CD164Ex5-f | TGAAATTTGGCTTTTGGTCTC |  |
| CD164Ex5-r | GAAGAGGAATCTCAGCAAAGC | 379 bp |
| CD164Ex6-f | AATGAATCACCAGTCCTTATTAATTC |  |
| CD164Ex6-r | GGACCATGAAACTTAGGATTGTT | 250 bp |
|  |  |  |
| **Sequencing primers (Copenhagen)** | **Sequence (5´- 3´orientation)** |  |
| CD164-Exon1-F | GGGAGCGTAGTCTCGGAGG |  |
| CD164-Exon1-R | GCCATGTTGCCGGAGTC |  |
| CD164-Exon2-F | TGATTGGAATTTCTTAGTAAACCAG |  |
| CD164-Exon2-R | TGATTGGAATTTCTTAGTAAACCAG |  |
| CD164-Exon3-F | TCTAAAGGGAGGATGGGAGAC |  |
| CD164-Exon3-R | AAACACCTAAAGTGGTTCCCC |  |
| CD164-Exon4-F | TGGCAATGCTTCAAACAAAG |  |
| CD164-Exon4-R | TCAAATGATTGCAGGTCAGG |  |
| CD164-Exon5-F | AACGTTAATTGCTGTGGTCC |  |
| CD164-Exon5-R | CCTAGTATTCTTGGGTGCATAGC |  |
| CD164-Exon6-F | TGGCAGAAATTGTAAAGGGC |  |
| CD164-Exon6-R | TGGCAAAATAGAGGCTCAGG |  |
| CD164-Exon7-F | CTCATTTGGCACGTTCTTCTC |  |
| CD164-Exon7-F | GGCTGTCATTTCAAAAGTGGA |  |
| CD164-Exon7-R | TCACTGTCTTCTAAGGCACTGTTC |  |
|  |  |  |
| **Sequencing primers for cDNA** | **Sequence (5´- 3´orientation)** |  |
| cDNA-f | CTAAACCCACAGTTCAGCCCTCC |  |
| cDNA-r | TCCCAAACATCCTATATGCATCCAT |  |
|  |  |  |
| **qPCR of transfected cell lines** | **Sequence (5´- 3´orientation)** |  |
| CD164common-F | CTACCTTTGATGCAGCCAGT |  |
| CD164common-R | TTACAGAGTGTGGTAATTTC |  |
| CD164wt-R | TTACAGAGTGTGGTAATTTCg |  |
| CD164mut-R | TTACAGAGTGTGGTAATTTCa |  |
|  |  |  |
| ***LightCycler assay for CD164 c.574C>T** | **Sequence (5´- 3´orientation)** |  |
| rs62436104_F | ACCTCACAACCTGTGCGA |  |
| rs62436104_R | TCACCAGTCCTTATTAATTCAATGG |  |
| Wildtype SP (probe) | GCAAATCTXIAAAGAACGAAATTACCACA--PH |  |
| ***This assay is also detecting neighbour SNP rs62436104** |  |  |
