# Supplementary material for: A Novel Locus Harbouring a Functional CD164 Nonsense Mutation Identified in a Large Danish Family with Nonsyndromic Hearing Impairment
Source: PLoS Genet. 2015 Jul 21;11(7):e1005386. doi: 10.1371/journal.pgen.1005386 (PMC4510537; doi:10.1371/journal.pgen.1005386)
Supplement: S4 Table — (DOCX) [file pgen.1005386.s009.docx]

| **Target region capture statistics** | **Individual IV-31** |
| --- | --- |
| Target size (bases on + strand) | 24,915,083 |
| Read lenght (bp) | 36 |
| Total number of reads | 90,069,516 |
| Mapped reads | 82,833,823 |
| Uniquely mapped reads | 64,554,391 |
| Uniquely mapped to target region | 40,085,147 |
| Average sequencing depth on target | 60.49 |
| Percentage of target covered ≥ 1X | 94.70 |
| Percentage of target covered ≥ 5X | 87.90 |
| Percentage of target covered ≥ 10X | 84.97 |
| Percentage of target covered ≥ 15X | 83.24 |
| Percentage of target covered ≥ 20X | 81.57 |
| Percentage of target covered ≥ 25X | 79.57 |
| Percentage of target covered ≥ 30X | 77.04 |
